# Coupling to octahedral tilts in halide perovskite nanocrystals induces phonon-mediated attractive interactions between excitons

**DOI:** 10.1038/s41567-023-02253-7

**Published:** 2023-11-09

**Authors:** Nuri Yazdani, Maryna I. Bodnarchuk, Federica Bertolotti, Norberto Masciocchi, Ina Fureraj, Burak Guzelturk, Benjamin L. Cotts, Marc Zajac, Gabriele Rainò, Maximilian Jansen, Simon C. Boehme, Maksym Yarema, Ming-Fu Lin, Michael Kozina, Alexander Reid, Xiaozhe Shen, Stephen Weathersby, Xijie Wang, Eric Vauthey, Antonietta Guagliardi, Maksym V. Kovalenko, Vanessa Wood, Aaron M. Lindenberg

**Affiliations:** 1 Department of Materials Science and Engineering, Stanford University, Stanford, CA USA; 2 Stanford Institute for Materials and Energy Sciences, SLAC National Accelerator Laboratory, Menlo Park, CA USA; 3 Department of Information Technology and Electrical Engineering, ETH Zürich, Zürich, Switzerland; 4 Department of Chemistry and Applied Biosciences, ETH Zürich, Zürich, Switzerland; 5 Empa-Swiss Federal Laboratories for Materials Science and Technology, Dübendorf, Switzerland; 6 Dipartimento di Scienza e Alta Tecnologia & To.Sca.Lab, Università dell’Insubria, Como, Italy; 7 Department of Physical Chemistry, University of Geneva, Geneva, Switzerland; 8 X-ray Science Division, Argonne National Laboratory, Lemont, IL USA; 9 Department of Chemistry and Biochemistry, Middlebury College, Middlebury, VT USA; 10 Chemistry and Materials Design Group, Department of Information Technology and Electrical Engineering, ETH Zürich, Zürich, Switzerland; 11 SLAC National Accelerator Laboratory, Menlo Park, CA USA; 12 Istituto di Cristallografia & To.Sca.Lab, Consiglio Nazionale delle Ricerche, Como, Italy; 13 Stanford PULSE Institute, SLAC National Accelerator Laboratory, Menlo Park, CA USA; 14 Department of Photon Science, Stanford University and SLAC National Accelerator Laboratory, Menlo Park, CA USA

**Keywords:** Quantum dots, Electronic properties and materials, Quantum dots, Electronic devices

## Abstract

Understanding the origin of electron–phonon coupling in lead halide perovskites is key to interpreting and leveraging their optical and electronic properties. Here we show that photoexcitation drives a reduction of the lead–halide–lead bond angles, a result of deformation potential coupling to low-energy optical phonons. We accomplish this by performing femtosecond-resolved, optical-pump–electron-diffraction-probe measurements to quantify the lattice reorganization occurring as a result of photoexcitation in nanocrystals of FAPbBr_3_. Our results indicate a stronger coupling in FAPbBr_3_ than CsPbBr_3_. We attribute the enhanced coupling in FAPbBr_3_ to its disordered crystal structure, which persists down to cryogenic temperatures. We find the reorganizations induced by each exciton in a multi-excitonic state constructively interfere, giving rise to a coupling strength that scales quadratically with the exciton number. This superlinear scaling induces phonon-mediated attractive interactions between excitations in lead halide perovskites.

## Abstract (web summary)

Time-resolved measurements show that coupling between electrons and phonons in lead halide perovskites can mediate attractive interactions between excitons, although the interaction strength depends on the specific material.

## Main

Lead halide perovskites (LHPs) have advanced to the forefront of materials research for a wide array of applications including optoelectronic devices (for example, in solar cells)^1,2^, near-unity quantum yield light sources^3^ and coherent single-photon emitters for quantum information processing^4^. Electron–phonon coupling (EP-coupling) plays a critical role in LHPs, expected to both enhance performance metrics in some cases (including polaronic protection of charge carriers)^5^ and limit them in others (for example, exciton coherence loss and broadened emission in perovskite nanocrystals (NCs))^6^.

**Fig. 1:**
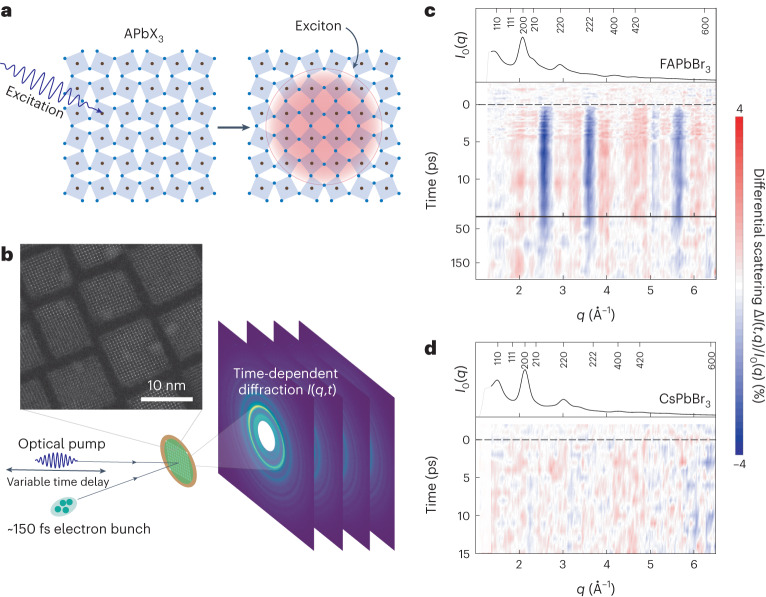
Time-resolved optical-pump–electron-probe measurements of formamidinium lead bromide nanocrystals. **a**, Illustration of a lattice reorganization of LHPs upon photoexcitation. **b**, Schematic of the experiment, including a high-resolution TEM image of FAPbBr_3_ NCs. **c**, Normalized time-resolved differential scattering of optically pumped FAPbBr_3_ NCs measured at 100 K with a pump fluence of 0.8 mJ cm^−^^2^. A strong and ultrafast reorganization of the FAPbBr_3_ lattice is observed upon photoexcitation. The solid black line is the equilibrium diffraction from the NCs, and marked Bragg peaks use the *hkl* of the cubic phase. **d**, Same as **c**, for CsPbBr_3_ NCs.

Although there has been extensive discussion of the coupling to the highest-energy longitudinal optical phonon (at about 17 meV in lead bromides) to interband transitions in these systems^7–9^, there is a growing appreciation for the importance of lower-energy optical modes (for example, in the range of about 2.5–12.0 meV in lead bromides)^10–12^, particularly in the hybrid lead halides, where their coupling can outweigh that of the high-energy longitudinal optical mode^13–16^. At the root of EP-coupling is a shift of the equilibrium atomic coordinates of the atoms in a material after a change of the electronic configuration (Fig. 1a). Although various time-resolved spectroscopies have shed light on the timescales of photoexcitation-induced lattice reorganization and the phonons involved^7,11,17,18^, the nature of the reorganization and therefore the mechanisms underlying the coupling remain unclear. Valuable insight can be provided through physical characterization of the inherent excited-state structural dynamics of these materials^19,20^. In principle, lattice reorganization can be directly measured through time-resolved diffraction. In a semiconductor NC, the size of which is comparable to or smaller than the exciton radius, such reorganization is expected to occur over the entire volume of the NC. Furthermore, NCs offer the possibility of exciting large numbers of excitons simultaneously within the same volume, which can enhance the magnitude of the lattice reorganization, facilitating its detection.

Here, we perform time-resolved, optical-pump–electron-diffraction-probe measurements to quantify the lattice reorganization occurring as a result of EP-coupling to the interband transition in formamidinium lead bromide (FAPbBr_3_, FA = CH5N_2_) NCs. We observe that excitons drive a reorganization of the Pb–Br sublattice towards higher symmetry, in contrast to the Fröhlich polaron picture in which one expects a decrease in overall symmetry as a result of lattice polarization. To explain this finding, we develop a deformation-potential EP-coupling model on the basis of the fact that in LHPs, the reduction of PbBr_6_ octahedra tilts drives a redshift renormalization of the bandgap^21–24^, which reduces the total energy of the excitons. We then use our model to extract EP-coupling strengths (Huang–Rhys factors) directly from the time-resolved measurements. Our findings provide an intuitive explanation for the origin of low-energy optical phonon coupling in LHPs and link the strong coupling to these modes in FAPbBr_3_ NCs to its locally tilted/disordered crystal structure^25,26^, which is found to persist down to cryogenic temperatures. Finally, the magnitude of the coupling strength is found to scale quadratically with the exciton number, inducing a phonon-mediated attractive interaction between excitons.

We performed measurements at the mega-electronvolt ultrafast electron diffraction facility (MeV-UED) at SLAC (Fig. 1b) on about 9.5 nm FAPbBr_3_ and CsPbBr_3_ NCs^27^, the sizes of which are comparable to estimated polaron (6–14 nm)^17,18,28^ and exciton (7 nm)^29^ diameters in lead bromide perovskites. Measurements are performed with pump fluences of 0.07–0.8 mJ cm^−^^2^. 400 nm pump photons are about 650 meV above the bandgap of the NCs and generate exciton densities *N*_ex_ of ~5–50 excitons per NC^30^. Experimental details are found in the Methods. From the measured time-resolved diffraction, *I*(*t*, *q*), we compute the differential scattering intensity as a function of time *t* and momentum transfer *q*

1 $${{\Delta }}I(t,q)=(I(t,q)-{I}_{0}(q))/{I}_{0}(q),$$

where *I*_0_(*q*) is the measured scattering of the sample in the absence of photoexcitation. The plot of *I*(*t*, *q*) at 100 K with 0.8 mJ cm^−^^2^ is shown in Fig. 1c and reveals a fully reversible reorganization of the FAPbBr_3_ lattice on photoexcitation, with large, fast changes in diffraction intensities at specific momentum transfers *q*. Under the same experimental conditions (100 K, 0.8 mJ cm^−^^2^), no lattice response in the CsPbBr_3_ NCs is discernible in the statistics of the measurement (Fig. 1d).

**Fig. 2:**
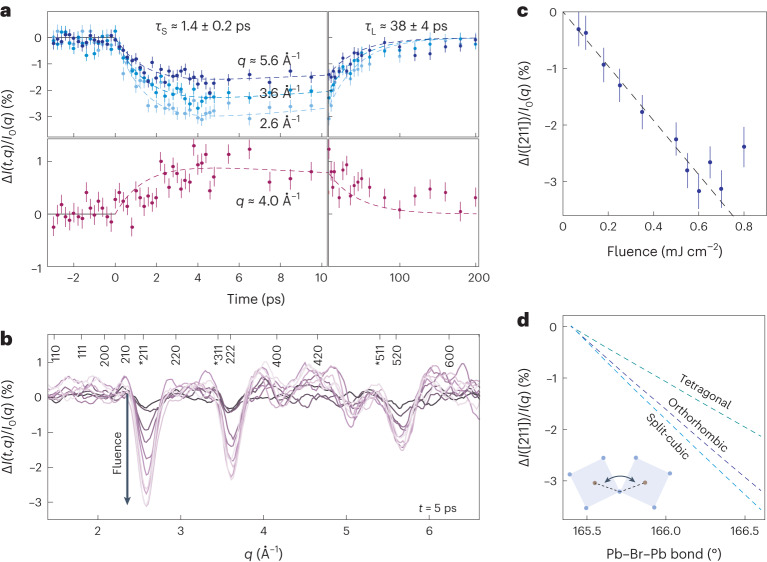
Picosecond lattice reorganization of FAPbBr3 NCs upon photoexcitation. **a**, Plot of the differential scattering at specific *q* with bi-exponential fit to the dynamics (dashed line; see Supplementary Fig. 2). **b**, Differential scattering at 5 ps for fluences ranging from 0.07 mJ cm^−^^2^ (darkest) to 0.7 mJ cm^−^^2^ (lightest). Main Bragg peaks are marked, along with weaker reflections corresponding to peaks that are minimized in the cubic phase and sensitive to octahedral tilting (*). **c**, Fluence dependence of the strength of the photoinduced lattice reorganization as extracted from changes in the 211 peak at about 2.6 Å^−1^. **d**, Simulated decrease in the intensity of the 211 reflection (about 2.6 Å^−1^) as a function of (primary) Pb–Br–Pb bond angle for a variety of LHP structures. Error bars in **a** and **c** represent 1*σ* uncertainty. Source data

The timescales of the dynamics of Δ*I*(*t*, *q*) are independent of *q*, as demonstrated in Fig. 2a, where Δ*I*(*t*, *q*) is plotted at specific *q* values. To quantify the dynamics, we fit the differential scattering with a bi-exponential function, exp[−*t*/*τ*_S_] − exp[−*t*/*τ*_L_], from which we extract the timescale for the onset of the lattice reorganization on excitation, *τ*_S_, and for the return of the lattice to equilibrium, *τ*_L_ (Supplementary Note 1 and Supplementary Fig. 2). The onset occurs on a timescale of *τ*_S_ ≈ 1.4 ps, irrespective of the pump fluence and temperature indicating a timescale intrinsic to the structural response of FAPbBr_3_ (see Supplementary Table 1). We note that this timescale is similar to the frequency of low-energy optical phonons in the lead bromide perovskites (0.6 THz)^31^. The lattice relaxes back to equilibrium on timescales of *τ*_L_ ≈ 30–50 ps, again with little discernible effect of pump fluence and temperature, which is in the range of measured multi-exciton decay rates in LHP NCs under similar excitation conditions^32,33^.

We rule out transient heating of the NCs^19^ as a cause of the observed lattice response as the measured timescales and magnitude of the lattice reorganization are not consistent with the trends expected due to thermal effects (Supplementary Note 2). The timescales rather point to a picture of lattice reorganization associated with the coupling of the lattice to the interband excitation of excitons. As the dominant EP-coupling in FAPbBr_3_ has been shown to be to lower-energy optical modes^11,16,34^, the observed lattice reorganization will be a distortion of the lattice along the normal coordinates of these phonons. We find that the magnitude of the differential scattering, Δ*I*(*t*, *q*), scales linearly with pump fluence (Fig. 2b), as highlighted in Fig. 2c for *t* = 5 ps and *q* = 2.6 Å^−1^. This finding indicates that the magnitude of the lattice reorganization is linearly dependent on the exciton number, *N*_ex_, which is also consistent with the fact that the timescale for the lattice to return to equilibrium *τ*_L_ is the same as that for multi-exciton recombination^32,33^.

As shown in Fig. 2b for *t* = 5 ps, the differential scattering is characterized primarily by strong reductions in the diffraction intensity at *q* values *q* *≈* 2.6, 3.6 and 5.6 Å^−1^, with a mild increase in the scattering at most other *q*. The largest differential feature at 2.6 Å^−1^ corresponds to the 211 peak (using cubic *hkl* indices). The magnitude of this peak is highly sensitive to the magnitude of Pb–Br–Pb bond angles in perovskite structures and is minimized in the cubic phase. In Fig. 2d, we plot the simulated intensity of the 211 peak as a function of the Pb–Br–Pb bond angle, where a linear proportionality is evident for a variety of low(er)-symmetry perovskite structures. Furthermore, the magnitude of the 211 peak is insensitive to distortions of the Pb–Br sublattice in which the Pb–Br–Pb bond angles remain invariant (Supplementary Note 3). The reduction of the 211 peak therefore indicates that excitons on the NCs drive a reduction of the magnitude of PbBr_6_ octahedra tilting, indicating an increase of the Pb–Br–Pb bond angle towards 180^∘^. The further differential scattering features at higher *q* (about 3.6 and 5.6 Å^−1^) cannot be accounted for only through a reduction in tilting. Further analysis of the differential scattering and discussion is provided in Supplementary Note 3.

Exciton–phonon coupling to the interband excitation of FAPbBr_3_ NCs therefore drives a structural lattice reorganization through reduction of Pb–Br–Pb bending. This finding is at odds with the simple picture of polar Fröhlich coupling, which would decrease lattice symmetry. Rather, we argue that these findings point to a deformation-potential-type EP-coupling to phonons that drives changes in the Pb–X–Pb bonding angles in the LHP.

**Fig. 3:**
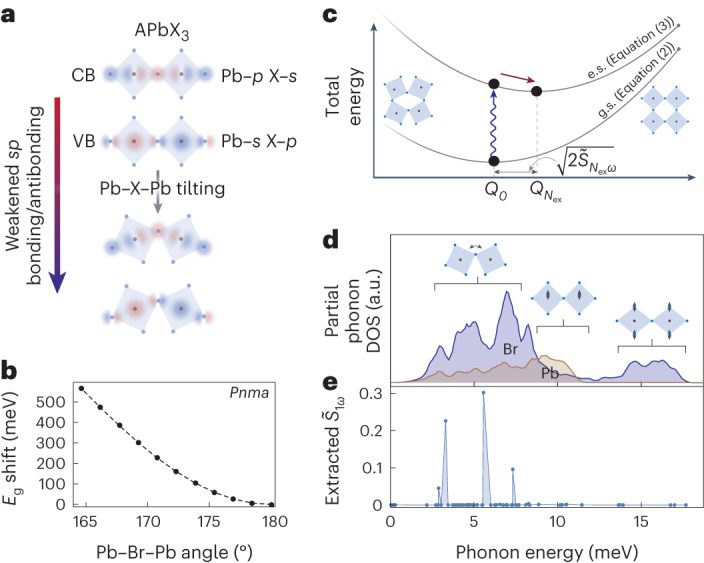
Model for EP-coupling resulting from distortions of the Pb–X sublattice. **a**, Cartoon schematic of the *sp*-bonding in the CB and VB of LHPs. **b**, Computed shift in the energy of the bandgap (*E*_g_) as a function of Pb–Br–Pb bending in orthorhombic *Pnma* CsPbBr_3_. **c**, Model for EP-coupling to phonons driving Pb–X octahedral tilting, where the presence of excitons shifts the minimum of the total energy of the excited state (e.s.) relative to the ground state (g.s.) towards the cubic phase. **d**, Computed partial phonon density of states (DOS) of CsPbBr3. Illustrations show the types of octahedral distortions driven by phonons in the specified ranges. **e**, EP-coupling strengths resulting from the coupling of the interband excitation of a single exciton to octahedral tilting in FAPbBr_3_ at 100 K, extracted from the magnitude of the measured changes in the 211 peak. Source data

It is known that Pb–X–Pb tilts and octahedral distortions affect the bandgap of LHPs^21–23^. Both the valence band (VB) and conduction band (CB) derive from *sp*-bonding of the Pb–X sublattice, with Pb–*s* and X–*p* antibonding about the VB-maximum and X-*s* and Pb-*p* antibonding about the CB minimum^24^. Any deviation of the Pb–X–Pb bond angles from 180^∘^ will decrease the *sp* coupling between atomic orbitals in both bands, reducing their bandwidth, thereby increasing the bandgap of the LHP (Fig. 3a). In Fig. 3b, we plot the renormalization of the bandgap, using bulk CsPbBr_3_ as a model system, where a blueshift occurs on increasing octahedral tilting. Although 180^∘^ Pb–X–Pb bonds minimize the bandgap, minimization of the lattice enthalpy determines the equilibrium structure, and LHPs frequently adopt lower-symmetry perovskite structures with finite Pb–X–Pb tilting^26,35^. A–X non-covalent interactions are thought to reduce the lattice-formation energies of the lower-symmetry polymorphs relative to the cubic phase^35–37^.

The strong bandgap renormalization occurring while bending the Pb–X–Pb angles means the phonons that drive these bends in the low-symmetry polymorphs couple to interband transitions. Provided finite tilting exists in the equilibrium phase of an LHP, in the excited state the coupling will drive a reduction in the magnitudes of the tilts, thus minimizing the exciton energy and increasing the lattice symmetry.

To illustrate this, we take a simple model assuming a single phonon with frequency *ω* and dimensionless normal coordinate *Q*, driving a *θ* bend (of the Pb–X–Pb bonds) in an LHP (Fig. 3c). In the absence of any excitation, the energy of the lattice, in the harmonic approximation, is given by

2 $${E}_{0}(Q)=1/2\hslash \omega {Q}^{2},$$

where ℏ is the reduced Planck constant and *Q* = 0 corresponds to the equilibrium phase with some finite tilt and bandgap *E*_g0_. We assume a first-order linear scaling of the bandgap along *Q* (so that ∂*E*_g_/∂*θ* ∝ ∂*E*_g_/∂*Q*; Fig. 3b and Supplementary Fig. 11). To first order, the energy of each exciton scales proportionally to the bandgap, and we write the total energy on exciting *N*_ex_ excitons as

3 $${E}_{{N}_{\text{ex}}}(Q)=\frac{1}{2}\hslash \omega {Q}^{2}+{N}_{\text{ex}}\left({E}_{\text{g}0}+\frac{\partial {E}_{\text{g}}}{\partial Q}Q\right).$$

This can be minimized to find the shift of the normal coordinate (magnitude of the lattice reorganization) in the excited state

4 $${Q}_{{N}_{\text{ex}}}={N}_{\text{ex}}\left(\frac{\partial {E}_{\text{g}}}{\partial Q}\right)/\hslash \omega ,$$

which scales linearly with the number of excitons, as observed in the experiments (Fig. 2c). The EP-coupling strength, typically referred to as the Huang–Rhys factor^38^, is given by

5 $${\tilde{S}}_{{N}_{\text{ex}}\omega }={[{Q}_{{N}_{\text{ex}}}]}^{2}/2.$$

In Supplementary Note 4, we provide a more detailed mathematical model that extends beyond the single-phonon assumption.

By computing and analysing the phonon density of states (Fig. 3d and Supplementary Note 4), we demonstrate that it is lower-energy optical phonons (about 2.5–8 meV) that couple to interband transitions as a result of Pb–X–Pb bond-angle distortions. For example, in the ideal orthorhombic *Pnma* structure, optical modes at 3, 6 and 7.5 meV drive the tilting in the Pb–Br LHPs. We can extract the EP-phonon coupling strengths of these modes to the excitation of a single exciton in the FAPbBr_3_ NCs from the MeV-UED results using the measured change in the 211 intensity (Fig. 3e and Supplementary Note 5). The strongest coupling of ${\tilde{S}}_{1\omega }$ ~0.3 to the 6 meV optical mode is in excellent agreement with that reported from low-temperature single-dot luminescence measurements, where a coupling to a 5 meV mode of about 0.15–0.35 was estimated for similarly sized NCs^16^. Couplings to the same modes calculated for equivalently sized CsPbBr_3_ NCs are more than an order of magnitude weaker (for example, ${\tilde{S}}_{1\omega }$ ~0.015 for the 6 meV mode; Supplementary Fig. 12), consistent with previous estimates of EP-coupling strengths in CsPbBr_3_ at low temperature^16,39^. The strong contrast in low-temperature EP-coupling strength in FA versus Cs explains why we observe a large lattice reorganization in FAPbBr_3_ and not in CsPbBr_3_ (Fig. 1c,d).

**Fig. 4:**
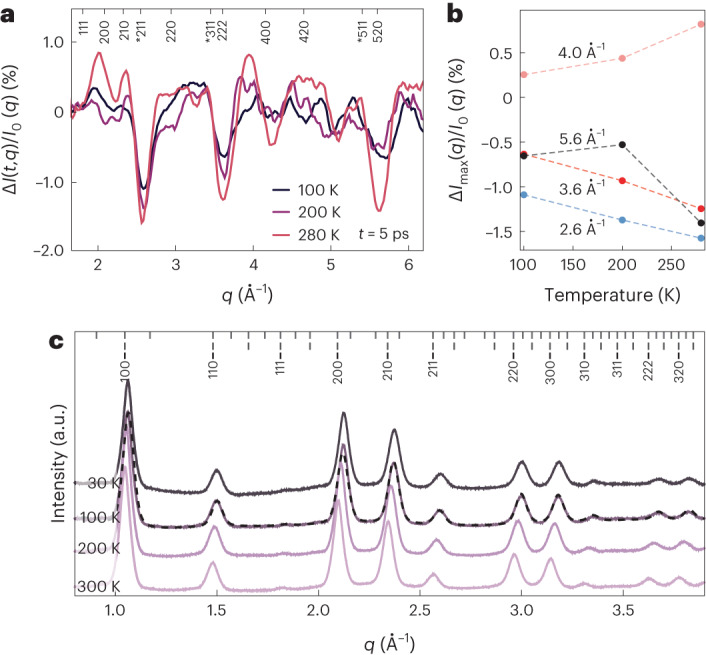
Enhanced and temperature-dependent EP-coupling in polymorphous FAPbBr_3_ NCs. **a**, Differential scattering measured on FAPbBr_3_ NCs at 100, 200 and 280 K with a fluence of 0.5 mJ cm^−^^2^. Bragg peaks are labelled as described in Fig. 2c. **b**, Plot of the maximum differential signal in **a** as a function of temperature at specific *q* values, indicating an enhancement with temperature of the photoinduced lattice reorganization. **c**, Temperature-dependent wide-angle X-ray total scattering data of FAPbBr3 NCs collected at 300–30 K. Ticks on the upper axis correspond to Bragg peaks of the orthorhombic (top), tetragonal (middle) and cubic (bottom) phases. The absence of characteristic superstructure peaks in the 1.6–2.0 Å^−1^ range, as well as the lack of notable changes in peak intensities among the *T*-dependent datasets, highlights the persistence of the polymorphic SC structure in the entire range of temperatures explored, as exemplified by the refined model fit shown by the dashed line for the 100 K scattering. Source data

To gain insight into the origins of the strong coupling to low-energy optical phonons in FAPbBr_3_, we consider its crystal structure and molecular/ionic orientations. Bulk FAPbBr_3_ has been reported to adopt distinct phases for specific temperature regimes in which it is orthorhombic below 137 K, tetragonal up to 262 K and cubic above 262 K (ref. ^40^). We repeat our time-resolved MeV-UED measurements at temperatures of 100, 200 and 280 K (Fig. 4a). Surprisingly, we find similar lattice response at all three temperatures, with an increase in the magnitude of the lattice reorganization at higher *T* (Fig. 4b). This finding implies non-zero magnitudes of octahedral tilting in the equilibrium structure even at 280 K. This is at odds with the assignment of a simple *Pm-3m* cubic perovskite structure with straight Pb–Br–Pb linkages but is consistent with FAPbBr_3_ NCs exhibiting disordered Br ions (and locally tilted Pb–Br–Pb angles) in an average-cubic phase^27^. In this case, photoexcitation reduces Pb–Br–Pb bending, pushing the system towards the archetypal *Pm-3m* cubic phase. This disordered phase has been described with the split-cubic (SC) perovskite model in which the Pb–Br–Pb angles are locally bent but lack any long-range order, making the average structure metrically and structurally cubic^41^. We note that in the SC structure, the 211 intensity is highly sensitive to the magnitude of the local Pb–Br–Pb bending (Supplementary Fig. 6).

We propose that the observed enhancement in the coupling of low-energy optical phonons to interband transitions in FAPbBr_3_ NCs is linked to the occurrence of such a disordered phase. However, this link would imply a persistence of disordered structure down to 0 K, as the strong coupling has been shown to persist at cryogenic temperatures^16,34^. To confirm this, we perform temperature-dependent X-ray total scattering measurements (at the MS-X04SA beamline of the Swiss Light Source) and find no indication of long-range ordering or any low-symmetry LHP phase as the temperature is decreased, with the disordered phase observed over the entire measured range (30–300 K; Fig. 4c). The origin of this disorder is likely a glassy state of the FA orientations^42^, with strong correlations between the local octahedral tilts and the actual orientation of the large FA ions of *mm2* symmetry in an ideal *m3m* symmetry site^43,44^. In Supplementary Note 6, we discuss several possible mechanisms that can enhance EP-coupling in the disordered phase and reproduce the observed increase in coupling with temperature, including phonon softening, sizeable entropic contributions to the free energy of the FAPbBr_3_ lattice and correlations between anharmonic FA reorientation and Pb–Br distortions^44,45^.

Finally, we consider the implications of the strong coupling. For this, we turn our attention back to the finding that the magnitude of the lattice reorganization is linearly dependent on the exciton number, *N*_ex_ (Fig. 2c and equation (4)), which indicates constructive interference of the lattice reorganization from each exciton. In this case, the EP-coupling strength depends quadratically on both the magnitude of the lattice reorganization and the exciton number ${\tilde{S}}_{{N}_{\text{ex}}\omega }\propto {N}_{\text{ex}}^{2}$ (equation (5)). This quadratic scaling of ${\tilde{S}}_{{N}_{\text{ex}}\omega }$ leads to massive reorganization energies, ${\lambda }_{{N}_{\text{ex}}}$, associated with multi-excitonic states. With the coupling extracted for the FAPbBr_3_ NCs (Supplementary Note 5), the reorganization energy of, for example, a 20-exciton state would be ${\lambda }_{{N}_{\text{ex}}}\propto {\sum }_{\omega }{\tilde{S}}_{1\omega }{N}_{\text{ex}}^{2}\hslash \omega \approx 2.8$ eV.

**Fig. 5:**
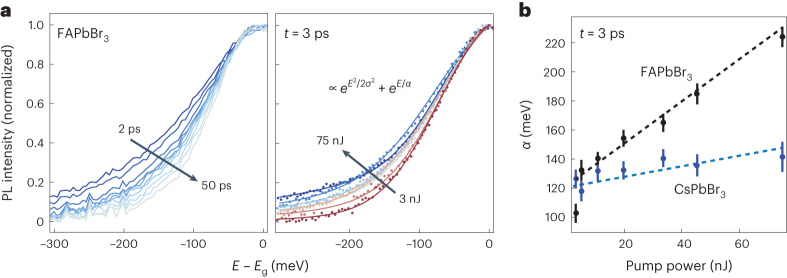
Time- and energy-resolved fluorescence. **a**, Plots of the normalized emission of FAPbBr_3_ NCs at different times for a 400 nm pump pulse of 75 nJ and at 3 ps for varying pump powers. The bandgap *E*_g_ is taken as the peak of the emission at large time delays. Strongly redshifted emission is observed at short timescales in the FAPbBr_3_ NCs. **b**, Plot of the energy scale of tails of the redshifted emission *α* in **a** as a function of pump power for FAPbBr_3_ and CsPbBr_3_ NCs. Error bars in **b** represent 1*σ* uncertainty from nonlinear least-squares fits to the luminescence spectra. Source data

This can be experimentally corroborated through measurement of the energy of photons emitted from the multi-excitonic state, as the emission energy of a single photon from an *N*_ex_ state will have a redshift of $2({N}_{\text{ex}}-1){\sum }_{\omega }{\tilde{S}}_{1\omega }\hslash \omega$ (which is about 265 meV for *N*_ex_ = 20) relative to the emission from the *N*_ex_ = 1 state. To investigate this, we perform time-resolved fluorescence upconversion photoemission spectroscopy (FLUPS) experiments. In these measurements, the photoluminescence (PL) from all NCs pumped by the Gaussian profile pump pulse is collected, and a large portion of the measured signal and the peak of the emission stem from emission from the large number of weakly pumped NCs at the periphery of the beam with *N*_ex_ ≤ 1 (Supplementary Fig. 16 and Supplementary Note 7). We therefore focus our attention on the low-energy tails of the emission. In the FAPbBr_3_ NCs, we observe a strongly redshifted contribution to the PL at short times, which increases with increasing fluence (Fig. 5a), as highlighted by an exponential fit to the tails of the emission at 3 ps, as shown in Fig. 5b. At the highest pump fluences, finite PL is observed all the way to the edge of the detector, at about 400 meV below the *N*_ex_ = 1 peak. This provides an independent confirmation of the large reorganization energies associated with the multi-excitonic states in the FAPbBr_3_ NCs.

Low-temperature quantification of EP-coupling in CsPbBr_3_ indicates far weaker coupling to the lower-energy optical modes^39^, consistent with our measurements (Fig. 1c,d) and estimates from theory (Supplementary Fig. 12). Recent room-temperature UED measurements have found lattice reorganizations qualitatively similar to those we observe in FAPbBr_3_ occurring in CsPbBr_3_ NCs from interband excitation at room temperature^46^. This indicates, as we have observed in FAPbBr_3_, an enhancement of deformation potential EP-coupling to the lower-energy optical modes with increasing temperature; we speculate this may be a result of finite softening and disordering of the nominally orthorhombic crystal structure of CsPbBr_3_ NCs already at room temperature, as observed in total X-ray scattering measurements^41^. Our room-temperature FLUPS measurements on CsPbBr_3_ NCs as a function of fluence exhibit redshifting of the emission with increasing fluence (Fig. 5b and Supplementary Fig. 18), but the effect remains far weaker in CsPbBr_3_ than FAPbBr_3_. This indicates that coupling to octahedral tilts, although enhanced at room temperature, remains weaker in CsPbBr_3_. In addition to the coupling to low-energy optical modes, coupling to the higher-energy optical mode (*ℏω* ≈ 17 meV) has been reported for CsPbBr_3_ NCs, with estimates for ${\tilde{S}}_{1\omega }$ ranging from 0.01 to 0.39 (refs. ^12,39^). The FLUPS measurements would only be consistent with the upper limit provided there is no constructive interference of the lattice reorganization (meaning no ${N}_{\text{ex}}^{2}$ scaling) stemming from the coupling of this mode.

The nonlinear scaling of ${\tilde{S}}_{{N}_{\text{ex}}\omega }$ to low-energy optical phonons implies an effective phonon-mediated attractive interaction between excitons, as the total reorganization energy associated with *N*_ex_ overlapping excitons, ${\sum }_{\omega }{\tilde{S}}_{1\omega }{N}_{\text{ex}}^{2}\hslash \omega$, is greater than that of *N*_ex_ spatially separated excitons, ${N}_{\text{ex}}{\sum }_{\omega }{\tilde{S}}_{1\omega }\hslash \omega$. This contributes to the total binding energy between excitons, *E*_B_ = *E*_B,C_ + *E*_B,P_, where *E*_B,C_ is the binding energy associated with Coulomb interaction between excitons and ${E}_{\text{B,P}}=({N}_{\text{ex}}^{2}-{N}_{\text{ex}}){\sum }_{\omega }{\tilde{S}}_{1\omega }\hslash \omega$ is the contribution from EP-coupling. For a biexciton in FAPbBr_3_, our extracted couplings (Fig. 3e) give an *E*_B,P_(FA) ≈ 14 meV. The weaker coupling to low-energy optical modes in CsPbBr_3_ implies a smaller contribution in *E*_B,P_(Cs). This is consistent with the 15 meV difference in reported biexciton binding energies in LHP NCs, where *E*_B_(FA) ≈ 25 meV (ref. ^16^) and *E*_B_(Cs) ≈ 10 meV (ref. ^47^).

These interactions can be expected to persist in bulk FAPbBr_3_, but the strength of deformation potential coupling and interactions between excitations may differ. In particular, the magnitude of deformation potential coupling scales with the volume of the excitation, $\tilde{S}\propto {V}^{-1}$, determined by the exciton/polaron radius or, in the case of NCs, restricted to at most the NC volume. Increased spatial confinement of the excitations will therefore increase the magnitude of the coupling. Furthermore, enhanced coupling due to surface-induced local tilting and phonon softening has been argued for CsPbBr_3_ NCs^48^. It is not clear, however, the extent to which this plays a role in the FAPbBr_3_ NCs, which maintain a locally tilted structure throughout the entire NC. Our measurements here were performed on about 9.5 nm NCs, considered to be in a weak confinement regime; reported size-dependent EP-coupling shows only a mild decrease of the deformation potential coupling from 9.5 nm to 16 nm NCs^16^, indicating only a slight decrease of the coupling in bulk FAPbBr_3_ compared to the values reported here. Finally, deformation potential coupling to Pb–X–Pb bends should be present for other LHP systems with differing compositions (other A-cations or X-anions) or dimensions (for example, two-dimensional (2D) LHPs), provided the VB, CB or both consist of *sp*-bonding of the Pb–X sublattice. For example, photoinduced straightening of Pb–I–Pb bends has recently been reported in 2D LHPs^49,50^. The strength of the coupling and interactions, and therefore their relevance, depends on the particular LHP system.

Although this attractive interaction between excitons will be short lived as a result of multi-exciton lifetimes (which are in the range 30–50 ps)^32,33^, equivalent deformation potential couplings and phonon-mediated effective attractive interactions are expected for both bare electrons and holes (Supplementary Note 4), which raises the possibility of correlated charge-carrier transport. Additionally, we find that the magnitude of the coupling increases with increasing temperature (Fig. 4b), which may enable the persistence of correlation effects at elevated temperatures.

## Methods

### NC synthesis and sample preparation

NCs capped with didodecyl dimethyl ammonium bromide ligands were prepared using previously described procedures for FAPbBr_3_ (refs. ^27,51^) and CsPbBr_3_ NCs^52^. For time-resolved electron-diffraction measurements, about two monolayers of NCs were deposited onto 75 mesh transmission electron microscope (TEM) grids with amorphous carbon support (Ted Pella 01802-f). TEM grid were first fixed using anticapillary tweezers, followed by deposition of 3 μl of NC solution in mesitylene with a concentration of about 1 mg ml^−1^. TEM images of the samples are given in the Supplementary Information.

### Optical-pump–electron-diffraction-probe measurements

We took measurements at the MeV-UED at SLAC, where the instrument is part of the Linac Coherent Light Source user facility. Details of the instrument have been reported previously^53,54^. A multipass-amplified Ti:sapphire laser with a repetition rate of 360 Hz provides ~150 fs full-width at half-maximum pulses of 800 nm photons. These pulses are split: one path generates electron beam pulses, and the other path is supplied to an optical parametric amplifier that generates the 400 nm optical-pump pulses. The generated electron bunches are accelerated to 3.7 MeV, resulting in ~10 fC pulses of ~150 fs width and 100 μm diameter at the sample. The delay between the ~500 μm optical-pump pulses and the electron probe pulses were adjusted by a translational stage in the optical pump’s beam path. *t* = 0 was calibrated through measurements on a bismuth thin film, and the *q* scale was calibrated from single-crystal diffraction on a thin film of gold. The diffracted electrons were measured by means of a red phosphor screen. The 2D diffraction data from the detector are azimuthally integrated into one-dimensional diffraction profiles. All measurements were performed on three identically prepared samples. Time scans at fixed fluence were performed with a complete randomization of the pump-probe delay times of the measurement, and a new position and/or sample was chosen for each time scan. Fluence scans were performed with randomization of the measured fluence at time delays of −3 ps (*I*_0_) and 5 ps. The measurement time for each fluence was adjusted to obtain similar statistics for each fluence. A new position on the same sample was chosen for each fluence measurement.

### DFT calculations

All density functional theory (DFT) calculations were performed using the Vienna Ab initio Simulation Package^55–58^, with projector augmented-wave (PBE.52) potentials^59,60^ and the GGA-PBE exchange-correlation functional, with a 520 eV cutoff and a 9 × 9 × 9 gamma-centred Monkhorst–Pack mesh^61^. The structural calculations (cell optimization, phonon calculations) were performed without spin-orbit coupling, which was then included for the final electronic structure calculations. The unit cell volume and primary tilt angle were optimized for the *Pnma* structure; the optimized structure is given in Supplementary Table 2. Calculations of the bandgap as a function of primary *Pnma* tilt were performed on structures in which only the tilts were modified and the unit cell volume was kept constant. The individual vacuum levels of computed bandstructures were shifted according to the mean of the lowest-energy Pb *d*-bands. For the calculation of gamma-point phonons and the phonon density of states, the atomic positions in the *Pnma* structure were optimized to a force convergence of 1 meV Å^−1^. Phonon density of states were computed using the Phonopy package^62^ with a 48 × 48 × 48 gamma-centred grid.

### Synchrotron WAXTS data collection and reduction

Wide-angle total scattering (WAXTS) measurements on FAPbBr_3_ NCs were performed at the MS-X04SA beamline of the Swiss Light Source (Paul Scherrer Institute, Villigen, CH)^63^ by drying a toluene colloidal suspension in a 0.5 mm borosilicate glass capillary of certified composition (Hilgenberg GmbH G50). The filled capillary was fastened with a special glue in a He cryostream to perform low-temperature measurements in the 300–30 K range. A beam energy of 22 keV was set, and the operational wavelength (*λ* = 0.563553 Å) was accurately determined using a silicon powder standard (NIST 640d, *a*0 = 0.543123(8) nm at 22.5 ^∘^C). Data were collected in the 0.4^∘^–130^∘^ 2*θ* range using a single-photon counting silicon microstrip detector (MYTHEN II)^64^. Background scattering from the sample holder and the empty glass capillary was independently collected under the same experimental conditions. Angle-dependent intensities corrections were applied to the raw data to account for sample attenuation due to absorption effects; sample absorption curves were determined using an X-ray tracing method^65^ and by measuring the transmitted beam from the filled capillary at room temperature; for the empty capillary, the X-ray attenuation coefficient was computed using its nominal composition. Angular calibrations were applied to the zero angle of the detector and to the *x*, *y* capillary offsets derived from the certified silicon powder standard (NIST 640d) using locally developed procedures. Background and (absorption-corrected) capillary scattering contributions were subtracted from the *T*-dependent sample signals.

### Time-resolved fluorescence upconversion photoemission spectroscopy measurements

The setup used is similar to that previously reported^66^. Excitation is provided by 100 fs at 400 nm pulses generated by doubling a portion of the output of a 1 kHz Ti:sapphire amplifier. Gate pulses of 1,340 nm are produced by an optical parametric amplifier, and a charged-coupled-device camera measures the upconverted spectra. Calibration was performed with secondary emissive standards.

## Online content

Any methods, additional references, Nature Portfolio reporting summaries, source data, extended data, supplementary information, acknowledgements, peer review information; details of author contributions and competing interests; and statements of data and code availability are available at 10.1038/s41567-023-02253-7.

### Supplementary information

Supplementary Information Supplementary Figs. 1–21, Discussion (Notes 1–7) and Tables 1 and 2.

### Source data

Source Data Fig. 2 Source data for Fig. 2a–c: time-dependent differential scattering data integrated about specific *q* values as indicated (**a**), power-dependent differential scattering profiles (**b**), maximum differential scattering intensity versus fluence (**c**).

Source Data Fig. 3 Source data for Fig. 3b,d,e: DFT-computed bandgap versus *Pnma* tilt angle for CsPbBr3 (**b**), computed partial phonon density of states of CsPbBr3 (**d**), extracted EP-coupling strengths in FAPbBr3 (**e**).

Source Data Fig. 4 Source data for Fig. 4a,c: temperature-dependent differential scattering profiles (**a**), temperature-dependent total X-ray scattering profiles of FAPbBr3 NCs measured at various temperatures (**c**).

Source Data Fig. 5 Source data for Fig. 5: time- and fluence-dependent luminescence measured on FAPbBr3 (**a**), extracted emission tail vs. fluence for FA- and CsPbBr3 (**b**).

## Data Availability

Source data are provided with this paper and are uploaded to the ETH Zurich Research Collection (10.3929/ethz-b-000626244). All other data that support the plots in this paper and other findings of this study are available from the corresponding authors on reasonable request.

## Code availability

The DebUsSy program suite is freely available at https://debyeusersystem.github.io.
