# Supplementary material for: Coupling to octahedral tilts in halide perovskite nanocrystals induces phonon-mediated attractive interactions between excitons
Source: Nat Phys. 2023 Nov 9;20(1):47–53. doi: 10.1038/s41567-023-02253-7 (PMC10791581; doi:10.1038/s41567-023-02253-7)
Supplement: Supplementary file 1 — Supplementary Figs. 1–21, Discussion (Notes 1–7) and Tables 1 and 2. [file 41567_2023_2253_MOESM1_ESM.pdf]

nature physics

Article

https://doi.org/10.1038/s41567-023-02253-7

Coupling to octahedral tilts in halide perovskite nanocrystals induces phonon-mediated attractive interactions between excitons

In the format provided by the authors and unedited

Index

Supplementary Note 1. Additional MeV-UED data S3
Table S1. S3
Figure S1. S4
Figure S2 S5
Figure S3. S5
Supplementary Note 2: Thermal Response of $FAPbBr_3$ S6
Figure S4. S6
Supplementary Note 3: Simulated Diffraction and Differential-Scattering Spectra in the 'Split-Cubic' model S7
Figure S5. S7
Figure S6. S10
Figure S7. S12
Figure S8. S13
Figure S9. S13
Figure S10. S14
Supplementary Note 4: Electron-Phonon Coupling Strengths Related to Octahedral tilting in Orthorhombic Lead Halide Perovskites S14
Figure S11. S16
Figure S12. S16
Supplementary Note 5: Extracting Electron-Phonon Coupling Strengths from Measured Lattice Reorganization S16
Figure S13. S17
Figure S14. S18
Figure S15. S18
Supplementary Note 6: Enhanced Coupling to Low Energy Optical Phonons in Polymorphous $FAPbBr_3$ S18
Supplementary Note 7: FLUPS Measurements on $FAPbBr_3$ and $CsPbBr_3$ NCs S19
Figure S16. S20
Figure S17. S20
Figure S18. S21
Figure S19. S21
Figure S20. S21
Figure S21. S21
Table S2. S22
References. S23

Supplementary Note 1: Additional MeV-UED data

Additional measured differential scattering maps are shown in **Fig. S1**. As discussed in the main text, we extract the timescale for the onset (τ_S) and relaxation (τ_L) of the lattice reorganization upon excitation by fitting the function $\sim\exp\left[-t/\tau_S\right] - \exp\left[-t/\tau_L\right]$ to the differential scattering. We fit to a weighted differential scattering intensity integrated over the entire measured q range,

$$\Delta I_w(t) = -\int dq\,\big(\Delta I(t,q)\big)w(q), \quad \text{(S1)}$$

using a q dependent weighting of $w(q) \equiv \int dt\Delta I(t,q)$. The computed $\Delta I_w(t)$ and fits for τ_S and τ_L are shown in **Fig. S2** for all pump fluences and temperatures for which time scans were measured.

In **Fig. S3** we plot the extracted differential scattering as a function of the azimuthal angle on the detector, from which an isotropic response of the lattice is evident. We therefore conclude that the observed lattice response is independent of the polarization of the pump beam.

Table S1. Extracted time constants for the lattice reorganization of $FAPbBr_3$ NCs. *Fit errors given in brackets*

Temperature (K)	Fluence (mJ/cm^2)	Onset τ_o (ps)	Relaxation τ_r (ps)
100	0.5	1.1 (0.2)	32 (4)
100	0.65	1.2 (0.2)	35 (4)
100	0.8	1.4 (0.2)	38 (4)
200	0.5	1.1 (0.2)	31 (5)
280	0.33	1.3 (0.3)	42 (8)
280	0.5	1.1 (0.2)	50 (10)

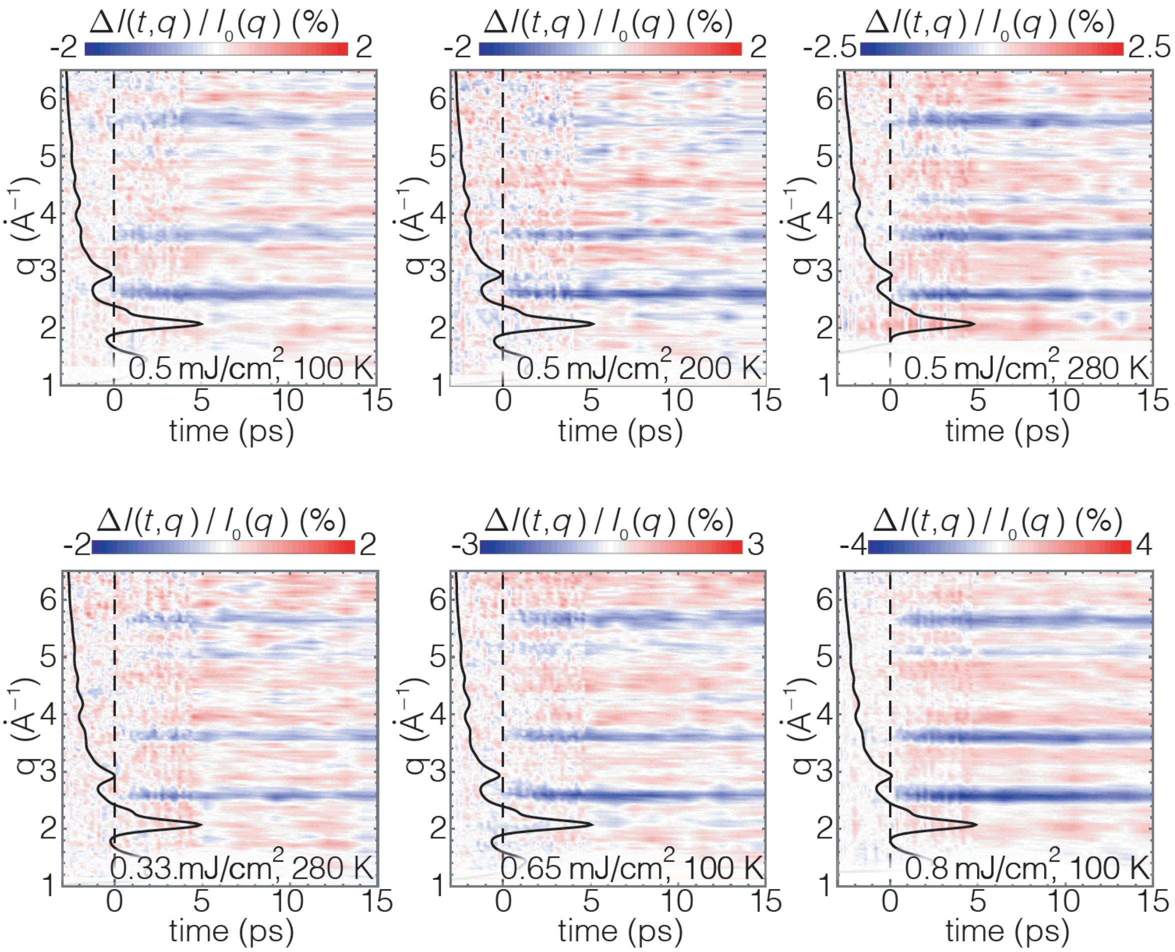

Figure S1. *Normalized time resolved differential scattering of optically pumped $FAPbBr_3$ NCs. Pump fluence and temperature of the measurement are shown on each map.*

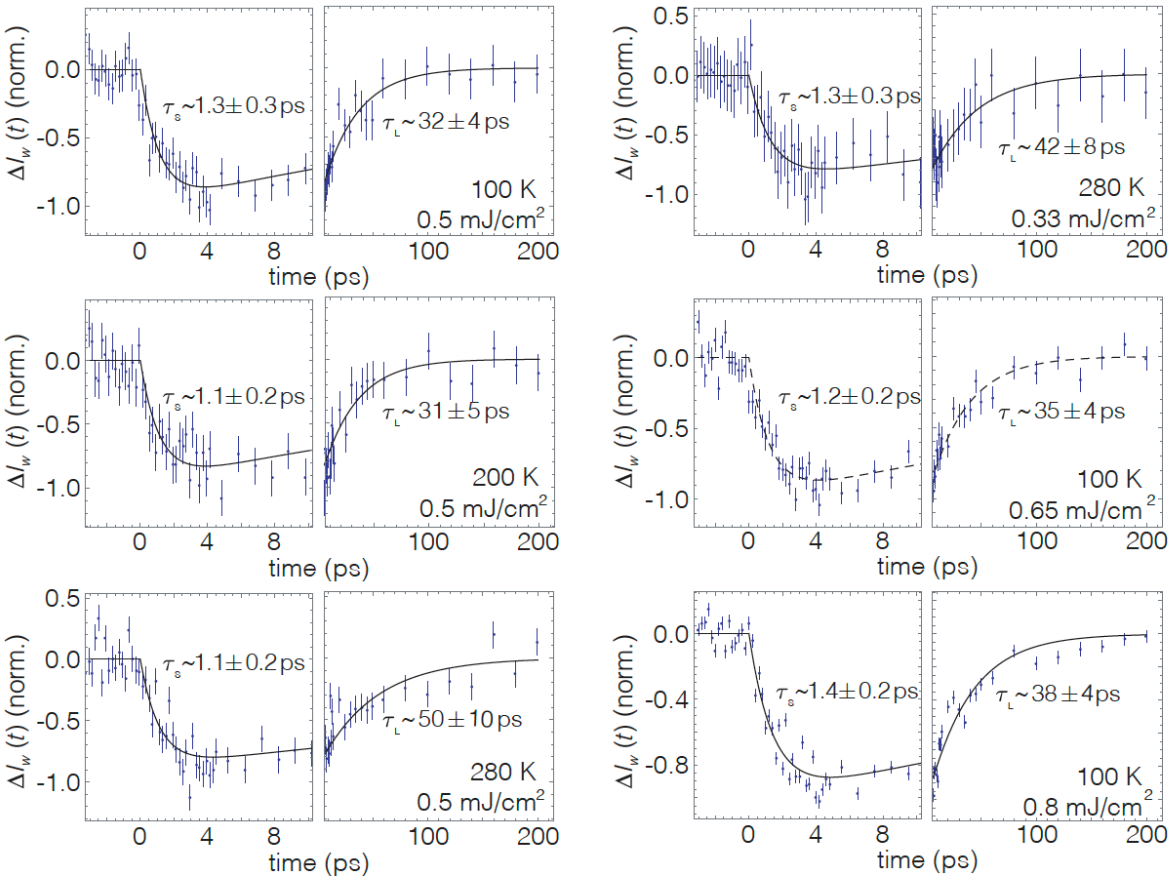

Figure S2. *Plots of the weighted differential scattering* (***eq. S1***) *along with corresponding biexponential fits. Pump fluence and temperature of the measurement are shown on each plot. Error bars represent 1σ uncertainty.*

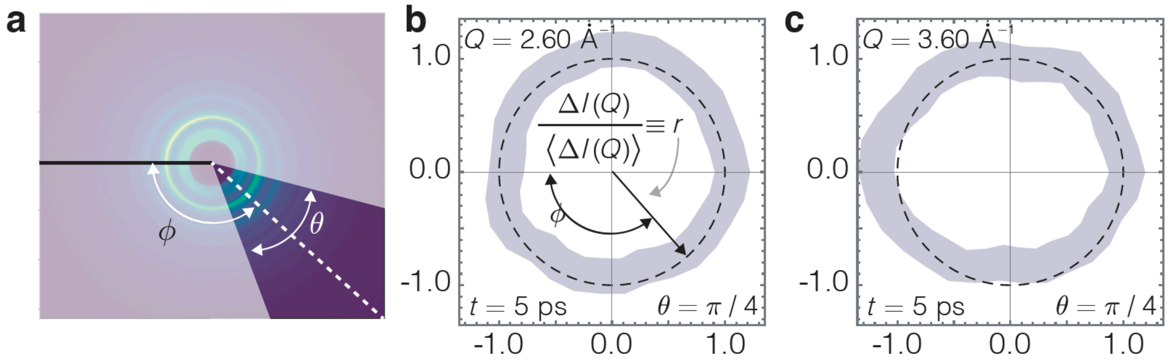

Figure S3. *Plot of the magnitude of the differential scattering at 5ps as a function azimuthal angle (ϕ in panel* ***a****), which indicates an isotropic response of the lattice. Data at 100 K with 0.8 mJ/cm^2 is shown here.*

Supplementary Note 2: Thermal Response of $FAPbBr_3$

It is important to rule out a transient heating of the NCs as a cause of the observed lattice response, as previously observed in $CsPbBr_3$ NCs.[1] In the experiments the $FAPbBr_3$ NCs are pumped with 400 nm photons, ~500 meV above the bandgap of the NCs, which enables the pumping of large exciton densities on the NCs. Initial carrier thermalization on sub-ps timescales will increase the temperature of the NCs (~22% of the final temperature increase from the excess ~650 meV energy), which is followed by heating stemming from the thermalization of Auger-excited hot-carriers majority (~78% of the temperature increase from the ~2.35 eV bandgap of the NCs).[2] At the highest fluence measured (0.8 mJ/cm^2), we estimate an average of ~50 excitons in the NC based on previously published estimates.[3] Using the reported[4] room temperature heat capacity for $FAPbBr_3$ of 1.69 MJ/m^{3}K and assuming the entire pumped energy, 50x3 eV = 150 eV, is converted to heat in the 9.5 nm NCs, we estimate a maximum temperature increase of ~17 K.

We performed temperature-dependent equilibrium measurements of the scattering of the $FAPbBr_3$ NCs on the same samples with the MeV-UED instrument. In **Fig. S4** we plot the differential scattering map (relative to the scattering at 100 K) as a function of temperature, $\Delta I(T,q) \equiv I(T,q) - I(100K,q)$. Heating from 100 to 280 K, we do see in the differential scattering some thermal expansion of the lattice, as well as a ~0.05% decrease in the 211 peak intensity. Critically, the maximum differential signal generated by a 180 K change in temperature (~0.1%) is over one order of magnitude smaller than the differential signals measured in the photo-response of the lattice. Considering we only expect at most a ~17 K increase in temperature in the photoexcited samples, the lattice thermalization response is completely negligible.

Additionally, we can also argue against thermalization induced effects from the extracted timescales of the lattice response. A majority of heating induced effects should set in on a time scale of the multi-exciton decay rate (Auger-heating), in stark contrast to our observation that the lattice reorganization decreases on this timescale.

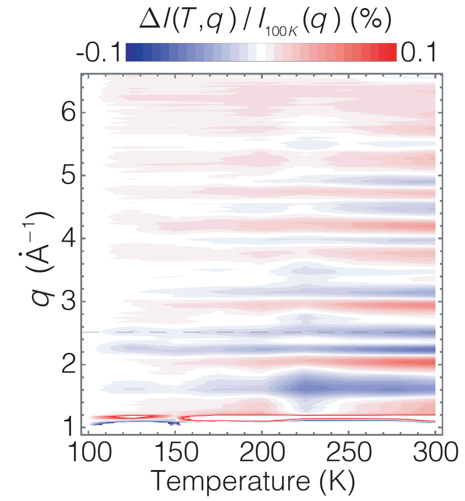

Figure S4. *Differential scattering map as a function of temperature, comparing the scattering at temperature T to the scattering at 100 K. Moderate lattice expansion is observed at elevated temperatures through a shifting to lower q of the main Bragg peaks, along with a ~0.05% decrease in the magnitude of the 211 peak (~ 2.6 Å^{-1}).*

Supplementary Note 3: Simulated Diffraction and Differential-Scattering Spectra in the 'Split-Cubic' model

The Debye Scattering Equation (DSE) Method

The DSE provides the average differential cross section (or the powder diffraction pattern) of a randomly oriented powder from the distribution of interatomic distances between atomic pairs, without any assumption of periodicity and order:[5,6]

$$I(q) = \sum_{j=1}^{N} f_j(q)^2 o_j^2 + 2\sum_{j>i}^{N} f_j(q) f_i(q) T_j(q) T_i(q) o_j o_i \frac{\sin(q d_{ij})}{(q d_{ij})} \quad (S2)$$

where $q = 4\pi\sin\theta/\lambda$ is the magnitude of the scattering vector, λ is the radiation wavelength, f_i is the atomic form factor of element i, d_{ij} is the interatomic distance between atoms i and j, N is the total number of atoms and T and o_i are the thermal atomic displacement parameter and the site occupancy factor associated to each atomic species, respectively. The first summation in the above equation includes the contributions of zero distances between one atom and itself and the second term (the interference term) the non-zero interatomic distances $d_{ij} = | r_i - r_j |$. This approach, compared to the conventional Rietveld method, takes advantage of the simultaneous modelling of Bragg and diffuse scattering and thus enables an appropriate description of the total scattering of samples when a limited extension of the coherent domains, the occurrence of structural defects and various kinds of compositional disorders are present, within an approach nearly free of phenomenological components. Complete DSE computation and analysis protocols have been implemented in an open source program package (the DebUsSy program Suite),[7] in which, thanks to computational tricks, such as reducing the number of interatomic distances by fully exploiting the crystal symmetry and using sampled interatomic distances, this computationally heavy approach becomes feasible.

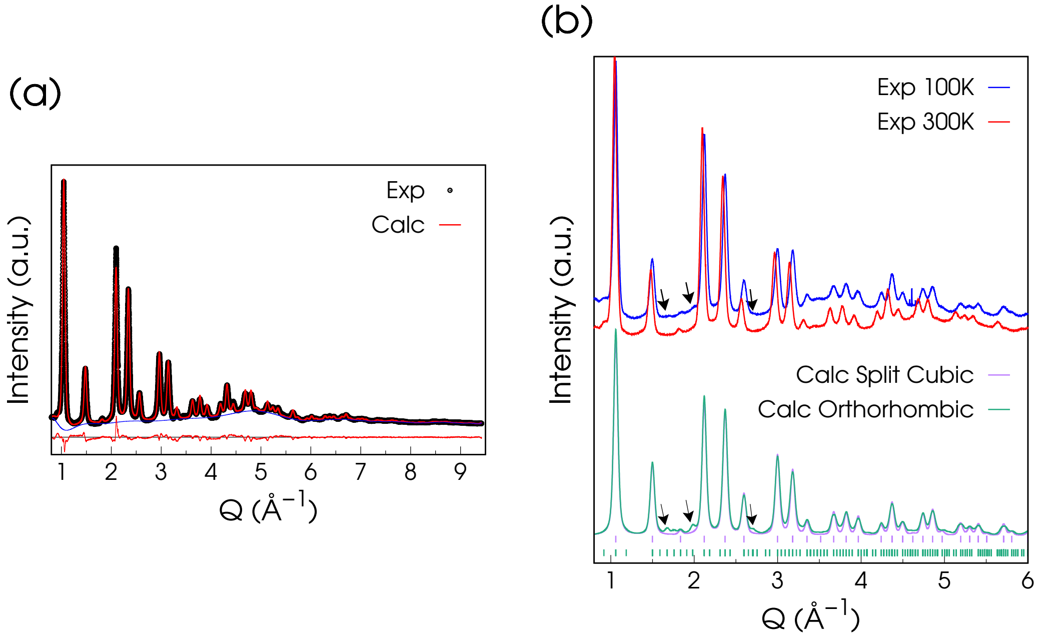

Figure S5. *a) Synchrotron X-ray total scattering data collected at 300K (black dots) and the Rietveld fit (red trace) for ~9 nm $FaPbBr_3$ nanocrystals, obtained by using a split-cubic structural mode.,[8] The blue line is the polynomial background implemented to model the*

nanocrystals diffuse scattering and some residual scattering from the He-cryostat shaft support used for the low temperature data collection (Figure 3 of the main text). The inset shows the refined $FaPbBr_3$ Pm-3m split-cubic structure, with local octahedra tilting ($\varphi = 11.4°$). b) Top: Synchrotron X-ray scattering data collected at 300K and 100K, showing the absence of superlattice peaks. Bottom: comparison between the split-cubic Pm-3m and orthorhombic Pbnm calculated patterns best fitting the 100K data. For the orthorhombic model, the Debye-Waller Pb and Br's B factors were constrained to the values in the split-cubic model (to make the comparison more appropriate), while lattice parameters and Br atomic coordinates were relaxed. This model describes an average orthorhombic structure throughout the entire nanocrystal volume but the corresponding distinctive features (highlighted by black arrows) vs the split-cubic model are not supported by the experimental data.

Atomistic models of $FAPbBr_3$ were built using cubic-shaped nanocrystals (NCs). A cubic unit cell parameter of 6.0756 Å, determined from experimental 100 K UED data of $FAPbBr_3$ NCs, was used for all simulations, as well as a cube-edge length of 7.1 nm (for a 8.8 nm equivalent spherical diameter). All DSE simulations were performed using sampled interatomic distances, encoded in the atomistic models, and using electron atomic scattering factors calculated by Mott-Bethe formulae. To account for the (low) angular resolution of UED experimental data, a pseudo-Voigt function was convoluted to the computed DSE patterns, using the following parameters: for the angle dependent full width at half maximum, fwhm(θ) = 0.6000 + 0.7062 tan(θ) + 0.6788 sec(θ); for the pseudo-Voigt mixing parameter, a constant η = 0.70 value.

Split-Cubic model for the $FAPbBr_3$ structure

As discussed in the main text and in Protesescu, L. *et al.*[8], $FAPbBr_3$ NCs exhibit a disordered structure with an average-cubic phase. In this disordered phase only local distortions of the Pb-Br sublattice are expected with no long-range order, e.g. locally tilted Pb-Br-Pb bonds. The complete structural reconstruction of glassy structures from scattering data are out of reach, and simplified structural models are often employed to interpret scattering results.

X-ray total scattering measurements on the $FAPbBr_3$ NCs, presented in Fig. **4** of the main text and in Fig. **S5**, lack any superstructure peaks beyond the peaks present for the cubic Pm-3m perovskite structure over the entire measured temperature range (30–300 K). The assumption of a simple Pm-3m perovskite structure, however, poorly reproduces the measured relative peak intensities. Weconclude , therefore, that the NCs exhibit a disordered phase, which, while on average cubic, has local distortions and multiple self-excluding positions of the Br ions.

This average-cubic disordered phase of perovskites has previously been described with the "split-cubic" structure model, presented, for example, in the seminal work of Protesescu, L. *et al*[8] for $FAPbBr_3$ NCs[8] and, for bulk powder, in the work of Hanusch *et al.*[9] In this model, Br anions, linked to two Pb ions within the 3D framework built by corner-sharing $PbBr_6$ octahedra, are disordered in four equivalent positions (with s.o.f. = 1/4, see **Fig. S6a**); these positions are displaced crosswise in directions orthogonal to the Pb···Pb edge, which nominally coincides with the straight Pb-Br-Pb atomic sequence of the archetypal undistorted Pm-3m cubic phase. With these displaced Br positions, the Pb-Br-Pb bond angles significantly deviate from the ideal 180° by φ° *(*180-φ being the resulting bending) The split cubic model alone provides an excellent fit to the measured diffraction pattern, as shown in Fig. **S5**.

Models used to simulate differential scattering

To analyze the measured differential scattering profiles (Fig. **2b** and **4a**), we move beyond the split cubic model, and consider all possible local distortions of the Pb-Br sublattice. We separate the local distortions into three classes of local displacements:

1) Displacements of the Br ions in directions perpendicular to the Pb-Br-Pb bonds (as in the split cubic model), inducing a tilt φ, or, equivalently, a Pb-Br-Pb bond angle.
2) Displacements of the Br ions in directions parallel to the Pb-Br-Pb bonds, labelled $\Delta_{Pb\text{-}Br}$.
3) Displacements of the lead ions from the nominal positions Δ_{Pb}.

We assume all such displacements are local, and while the displacements of each ion in each case are assumed to be of equal magnitude, the direction of the displacements is chosen stochastically (see figure **S6b**). In the data analysis presented here, we restrict displacements to the (x,0,0), (0,y,0), or (0,0,z) directions.

Correspondence between the 211 intensity changes and Pb-Br-Pb bending

In figure **S6c** we plot the differential diffraction profile measured with UED compared to the simulated differential profile assuming a photoexcited decrease in the Pb-Br-Pb bond angle (with increasing φ) There is a strong enhancement of the 211 peak as φ increases.
In figure **S6d** we plot the simulated differential scattering of the model with varying $\Delta_{Pb\text{-}Br}$, i.e. with displacements of the Br ions in direction parallel to the cell edge (the Pb···Pb vector, note that here the tilt angle φ is held fixed). With these displacements, there is negligible change in the relative intensity of the 211. Similarly, small and negligible changes to the 211 peak occur with Pb off centering, Δ_{Pb}.
We can therefore conclude that the relative intensity of the 211 peak can be used to quantify the magnitude of the displacement of the Br ions in the direction perpendicular to the cell edge, and, therefore, we take this intensity change as a proxy for the φ angle, and the (180- φ) Pb-Br-Pb bending, changes. The measured strong photoinduced reduction of the 211 peak, therefore, indicates a strong reduction of φ, as discussed in the main text.

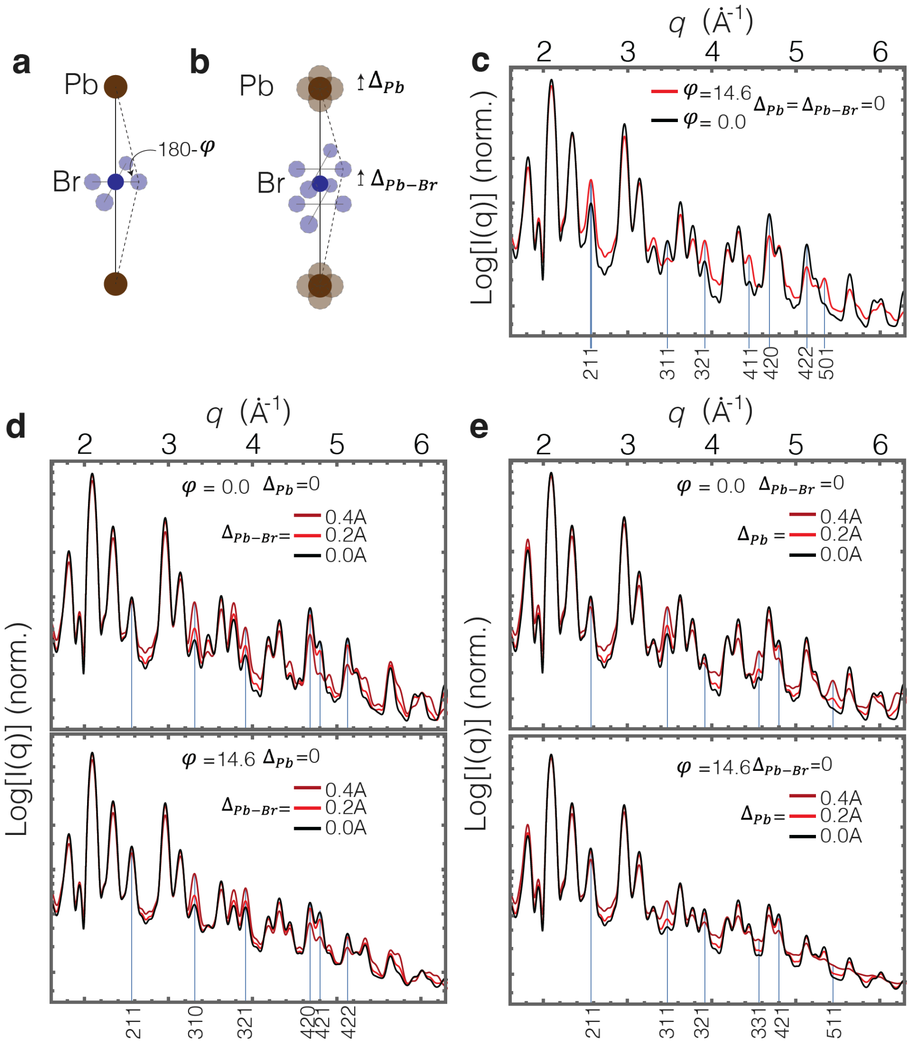

Figure S6. *a) diagram of the split-cubic model; b) diagram of the split-cubic model including displacements of the Br ion parallel to the Pb...Pb vector (Δ_{Pb-Br}) and Pb displacements (Δ_{Pb}). c) plot of the simulated electron diffraction profile for the split cubic model with φ=0 and φ=14.6°, with Δ_{Pb-Br} = Δ_{Pb}=0. d) simulated diffraction profile for the model with varying Δ_{Pb-Br}. e) simulated diffraction profile for the model with varying Δ_{Pb}.*

Negative differential scattering at 3.6 $Å^{-1}$

The second largest feature observed in the experimental differential scattering profile occurs at q ~ 3.6 $Å^{-1}$, which is approximately the position of the 222 peak. However, there are no distortions of the Pb-Br sublattice which selectively downsize the relative intensity of the 222 peak relative to the other high symmetry peak, *e.g.* 200. This can be seen in **Fig. S6**, where the change in the intensity of the 222 peak follows closely that of the 200 for all possible distortions. This implies that the observed negative differential peak observed at q ~ 3.6 $Å^{-1}$ is not a result of a strong reduction of the 222, as this would contradict the slight enhancement observed for the 200. This feature is thus likely a result of a reduction of the other Bragg peaks close to the 222, or strong reduction of the diffuse scattering about the 222 peak. This feature remains, so far, uninterpreted.

Simulated differential scattering profiles

In **Fig. S7** we plot the simulated differential scattering profiles for the model described above, using the q resolution of the UED experiments compared to the measured differential scattering.

A photoexcited change in φ (**S7a**) gives a good agreement only at low q, as it correctly reproduces the strong reduction in the 211 peak, but not that at peaks falling near 3.6 and 5.6 $Å^{-1}$.

We next considered a photo-induced increase in Δ_{Pb-Br} (**S7b**) in addition to a decrease in ϕ, which would result from a Fröhlich-type coupling to the high energy optical phonons in lead-bromides (~15-20 meV, see figure **3d** of the main text). The simulated differential spectra are at complete odds with that measured, showing strong positive differential peaks in the 3.6 and 5.6 $Å^{-1}$ regions.

Conversely, assuming a photoinduced decrease in nominal Δ_{Pb-Br} (a regularization of the Pb-Br octahedra assuming finite distortions of them in the equilibrium structure), gives closer agreement (**S7c**). In this case, strong negative differential features appear close in q to the experimentally observed features. Similarly, if a nominal off-centering of the Pb ions in the equilibrium structure exists, (Δ_{Pb}), a photoinduced reduction of this off-centering leads to strong negative differentials close to the experimentally measured features at ~ 3.6 and 5.6 $Å^{-1}$ (**S7d**).

In the $FAPbX_3$ species, the relatively large FA molecules will make the displacements of the halide ions sticking out in the A-cation cages probably correlated. As we discuss in the main text, we speculate that photoexcitation may also induce slight reorientations of the FA molecules, facilitating reduction of the $PbBr_6$ octahedra tilts. Therefore, correlations in the photoexcited displacements of the Br ions within the A-cation cages (an effect that our model cannot capture), cannot be ruled out. More satisfactory fits to the differential spectra could result from considering such correlations, but would require a more detailed model for the equilibrium structure of the glassy $FAPbBr_3$, which, presently, is not available.

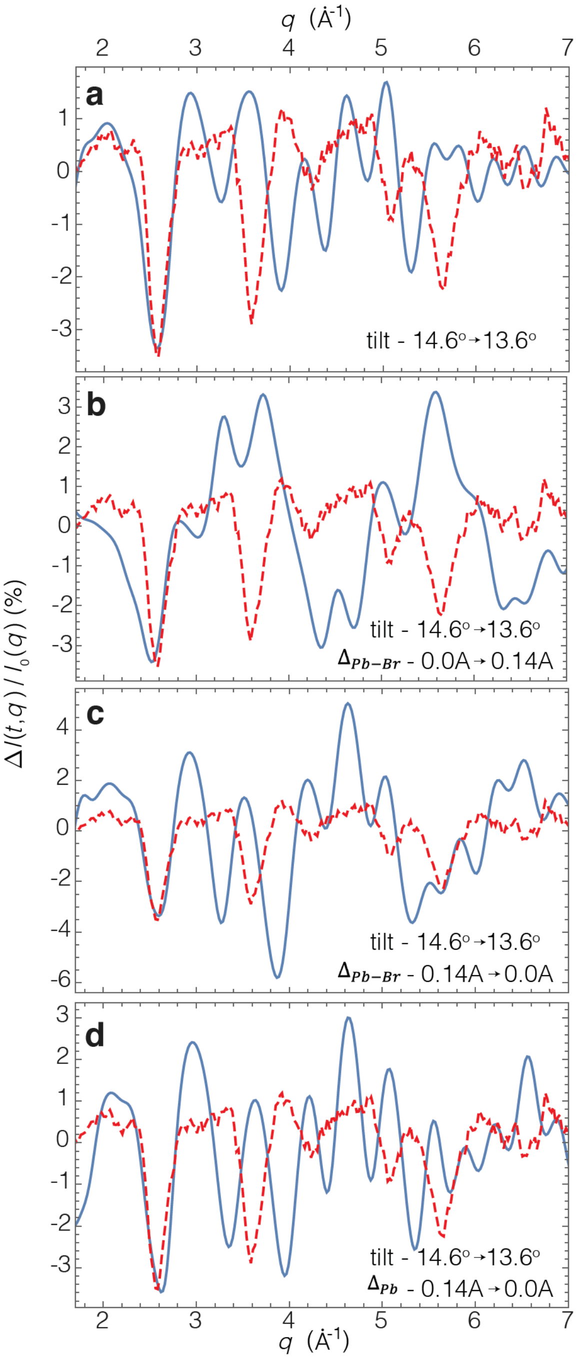

Figure S7. *Experimental differential scattering curve at 100K (red trace) vs simulated differential electron diffraction patterns (blue lines) using the model described above for FAPbBr₃ cubic shaped nanocrystals, assuming photoexcitation induced a) decrease in tilt, b) decrease in tilt and increase in* Δ_{Pb-Br}*, c) decrease in tilt and* Δ_{Pb-Br}*, d) decrease in tilt and* Δ_{Pb}*.*

<u>*Discussion*</u>

While it is not possible from the experiments to extract the precise photo-excited distortion of the Pb-Br sublattice beyond a reduction of tilting, we can use theory to elucidate possible mechanisms.

We first consider displacements of the Pb ions from the center of the octahedra, Δ_{Pb}. In **Fig. S8** we plot the shift in the bandgap as a function of Δ_{Pb}. Similar to the tilt dependence (**Fig. 3b**

of the main text) the bandgap is minimized when the Pb ions are located at the center of the octahedra. This result can be explained using the same argumentation employed to explain the tilting dependence; that the magnitude of sp bonding and antibonding in the CBM and VBM will be maximized when the Pb ions are centered.
In the idealized Pm-3m structure, there will be no coupling to phonons which drive Δ_{Pb} within the structure, as $\partial E_g/\partial\Delta_{Pb}|_{\Delta_{Pb}=0} = 0$. Finite Pb-off-centering has been shown to be intrinsic in the equilibrium structure of $FAPbBr_3$ (Δ_{Pb} > 0).[11] Given this, there will be finite coupling to phonons which drive Δ_{Pb}, and there will be a photoexcited decrease in the magnitude of Δ_{Pb}. Analysis of the phonon density of states indicates that these phonons should occur over an energy range of ~9-13 meV (see **Fig. 3d** of the main text).

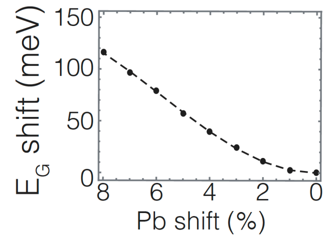

Figure S8. *Plot of the bandgap shift as a function of Pb displacement (Δ_{Pb}), the x-axis is in % of the nominal Pb-Br bond length*

Next we consider the coupling of the high energy LO phonon, ~17 meV. The high energy optical phonons in the energy range 15-20 meV drive tetragonal-distortions of the octahedra, displacing Br ions in the lattice along directions parallel to the Pb-Br-Pb bonds (see **Fig. 3d** of the main text). In our structural model above, these phonons, including the high energy LO, will thus drive changes in Δ_{Pb-Br}. In **Fig. S9** we plot the bandgap as a function of the phonon normal coordinate of the high energy LO mode, Q_{LO}. A very weak dependence is observed for small Q_{LO}. In the ideal Pm3m structure with perfectly regular octahedra, the coupling to this mode is thus expected to be 0. However, if there are equilibrium distortions of the octahedra, the coupling become finite, and increases in magnitude as the magnitude of nominal Δ_{Pb-Br} increases. Contrary to the coupling to tilting and Pb-off centering, in this case, EP-coupling to the LO will drive an increase in the octahedral distortions in the excited state. Our simulations assuming a photoexcited decrease in Δ_{Pb-Br} which somewhat qualitatively matches some of the high q differential spectra (**Fig. S7c**), is at odds with the theory. Conversely, we have seen that a large photoexcited increase of Δ_{Pb-Br} gives a differential spectrum that dramatically disagrees with the measured one (**Fig. S7b**).

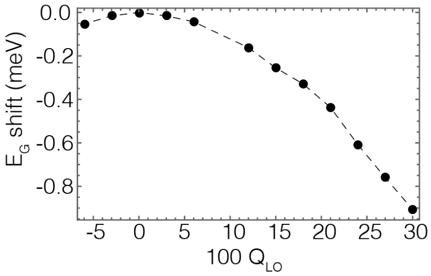

Figure S9. *Plot of the bandgap shift as a function of the shift of the normal coordinate of the highest energy LO phonon, Q_{LO}*

A coupling of this mode to interband excitation of a single exciton of S_{LO} ~0.05 has been extracted from single dot luminescence spectra on ~9 nm $FAPbBr_3$ NCs,[12] meaning a shift in $Q_{LO} = \sqrt{2S_{LO}} = 0.25$. From the LO phonon eigenvectors calculated for Pnma $CsPbBr_3$, a shift

in $Q_{LO} = 0.25$ corresponds to a displacement of the Br ions with $\Delta_{Pb-Br} = 0.006$ Å. Now, assuming the coupling of this mode also scales quadratically with the exciton number, $S_{LO,N} = N^2 S_{LO}$, or equivalently, $Q_{LO,N} = N Q_{LO}$, we would expect for ~40 excitons a shift of $\Delta_{Pb-Br}=$ 0.24 Å. Considering the plot in **Fig. S7b**, a shift of $\Delta_{Pb-Br}=$ 0.24 Å (twice that shown in the figure) would be detectable in the experiment, and would give a dramatically different differential signal to that measured. We therefore conclude that the coupling to the high energy LO mode does not scale quadratically with exciton number, in agreement with the FLUPS results of the main text. This lack of N^2 scaling would also explain the lack of the large positive differential signals expected to occur as a result of the increased Δ_{Pb-Br} (**Fig. S7b**).

Supplementary Note 4: Electron-Phonon Coupling Strengths Related to Octahedral tilting in Orthorhombic Lead Halide Perovskites

Assuming regular Pb-X_6 octahedra, the *Pnma* orthorhombic phase of a LHP is completely determined by specifying the Pb-X-Pb angle, the unit cell volume V or Pb-X bond distance, as well as two scalars, x_3 and z_3, specifying the shift of the A-site cation away from its position in the cubic phase.[13] One can write the x, y, and z coordinates (index α) of each atom within the unit cell (index i) as a vector $\boldsymbol{r}_{i\alpha}(\varphi, V, x_3, z_3)$. The distortion of the atomic positions within the unit cell between two *Pnma* structures with different primary tilts, φ and φ', can then be defined as $\boldsymbol{D}_{i\alpha} = \boldsymbol{r}_{i\alpha}(\varphi', V', x_3', z_3') - \boldsymbol{r}_{i\alpha}(\varphi, V, x_3, z_3)$. Here, we consider the case of distortion of the structure from the orthorhombic phase towards the cubic phase at fixed unit cell volume, $\boldsymbol{D}_{i\alpha}(\gamma) = \boldsymbol{r}_{i\alpha}((1-\gamma)\varphi, V, (1-\gamma)x_3, (1-\gamma)z_3) - \boldsymbol{r}_{i\alpha}(\varphi, V, x_3, z_3)$, using a parameter $0 \le \gamma \le 1$, where $\gamma = 0$ corresponds to the orthorhombic phase and $\gamma = 1$ to the ordered *Pm3m* cubic phase. We can then express $\boldsymbol{D}_{i\alpha}(\gamma)$ in terms of shifts along the normal coordinates of the $q = 0$ phonon modes of the orthorhombic phase, with frequencies ω_n and mode eigenvectors $\boldsymbol{w}_{n,i\alpha}$,[14]

$$Q_{\omega_n}(\gamma) = \sqrt{\frac{N_U}{4}} \sum_{i,\alpha} \frac{\sqrt{m_i \hbar \omega_n}}{\hbar} \boldsymbol{w}_{n,i\alpha} \boldsymbol{D}_{i\alpha}(\gamma), \quad (S3)$$

where m_i is the mass of atom i and N_U is the number of $APbX_3$ units over which the distortion occurs (the factor of 4 comes from the fact that the orthorhombic unit cell contains 4 $APbX_3$ units). We define the shift of the normal coordinates corresponding to the distortion between the orthorhombic and cubic phase as $Q_{\omega_n,c} = Q_{\omega_n}(1)$. We find that the $Q_{\omega_n}(\gamma)$ are linear in γ (or equivalently, linear with respect to φ, see **Fig. S10a**), so we can write $Q_{\omega_n}(\gamma) = \gamma Q_{\omega_n,c}$.

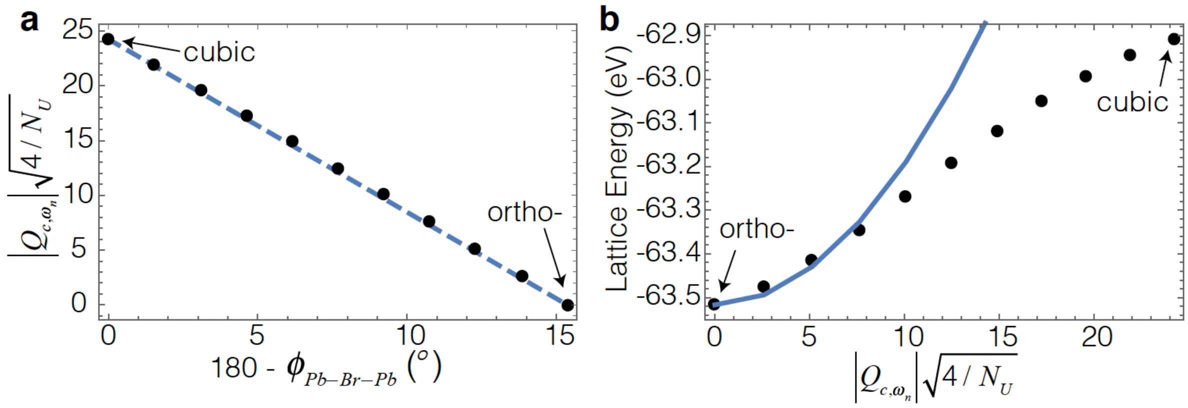

Figure S10. *a) Plot of the norm of the Q_{ω_n} as a function of Pb-Br-Pb angle, along with a linear fit (dashed line). b) Plot of the lattice formation energy (per Pnma unit cell) as a function of the norm of the Q_{ω_n} which is approximately quadratic for small Q_{ω_n}.*

For small deformations ($\gamma > 0$) from the orthorhombic structure upon the photoexcitation of N_{ex} excitons, we approximate the total energy as

$$E_{tot}(\gamma) = E_0 + \sum_n \frac{1}{2}\hbar\omega_n\left(\gamma Q_{\omega_n,c}\right)^2 + N_{ex}\gamma \left.\frac{\partial E_{ex}}{\partial \gamma}\right|_{\gamma=0} + \cdots . \ (S4)$$

Here E_0 is the total energy of the unexcited orthorhombic phase, the second term is the increase in formation energy of the distorted structure which is quadratic in $Q_{\omega_n,c}$ for small γ (see **Fig. S10b**), and the third term is the decrease in energy of the N_{ex} excitations relative to the orthorhombic phase, where $E_{ex}/\partial\gamma$ is the derivative of the excitation's energy with respect to γ (see **Fig. S11**). As in the main text, we assume here that energy of each exciton scales proportionally to the bandgap. N_U appearing in **eq. S3** is the number of $APbX_3$ units over which the N_{ex} excitations are localized, and we assume that the cubic distortion occurs uniformly over this volume. There can be additional terms, such as exciton binding energies, carrier-carrier interaction etc., however we assume here that these terms scale negligibly with γ, and do not write them out explicitly. We can then find the value for γ which minimizes eq. S4, γ_m, by solving $\partial E_{tot}/\partial\gamma = 0$,

$$\gamma_m = \frac{N_{ex}\left.\frac{\partial E_{ex}}{\partial \gamma}\right|_{\gamma=0}}{\sum_n \hbar\omega_n Q^2_{\omega_n,c}} . (S5)$$

The electron phonon coupling strength resulting from octahedral tilting can then be calculated by its definition

$$\tilde{S}_{\omega_n} = \frac{1}{2}\left(\gamma_m Q_{\omega_n,c}\right)^2 = \frac{1}{2}\left(\frac{N_{ex}\left.\frac{\partial E_{ex}}{\partial \gamma}\right|_{\gamma=0}}{\sum_n \hbar\omega_n Q^2_{\omega_n,c}}\right)^2 Q^2_{\omega_n,c} . (S6)$$

Both the magnitude of the cubic distortion (γ_m) and the coupling $\tilde{S}_{\omega_n}$ will depend on the number of unit cells over which the excitation is confined (both being proportional to N_U^{-1}). The polaron diameter, d_p, resulting from coupling to a phonon of frequency ω is given by[15]

$$d_p \sim 2\sqrt{\frac{\hbar}{m_{ex}\omega}}, (S7)$$

where m_{ex} is the effective mass of the excitation (i.e. electron, hole, or exciton effective mass). Taking the $CsPbBr_3$ exciton effective mass as $0.1m_e$,[16] we can estimate the lower limit of the polaron diameter by computing d_p for coupling to the ~17 meV high energy LO phonon, which gives ~14 nm. For $CsPbBr_3$ NCs, this is larger than the NC, and we can assume a polaron volume equal to the volume of the NC. In **Fig. S12b** we plot the computed $\tilde{S}_{\omega_n}$ for a single exciton on a $CsPbBr_3$ NC.

In **Fig. S11c,d** we plot the shift of the VMB and CBM as a function of octahedral tilt. An increase in the VBM and decrease in the CBM with increasing tilt is apparent, and the **eq. S6** can be used to compute the coupling of a bare charge, using $\partial E_h/\partial\gamma$ ~ -447 meV for holes and $\partial E_e/\partial\gamma$ ~ -480 meV for electrons assuming a fixed unit cell volume. These values suggest phonon-mediated attractive electron-electron and hole-hole interactions. We note that to arrive at the values of $\partial E_e/\partial\gamma$ and $\partial E_h/\partial\gamma$, the absolute position of the bands for the independent DFT calculations are aligned to a reference, which always carrier the risk of a systematic error in the extracted position of the bands. We used here the lowest 16 non-bonding Pb d-bands, occurring ~17.3 eV below the VBM as the reference for each calculation.

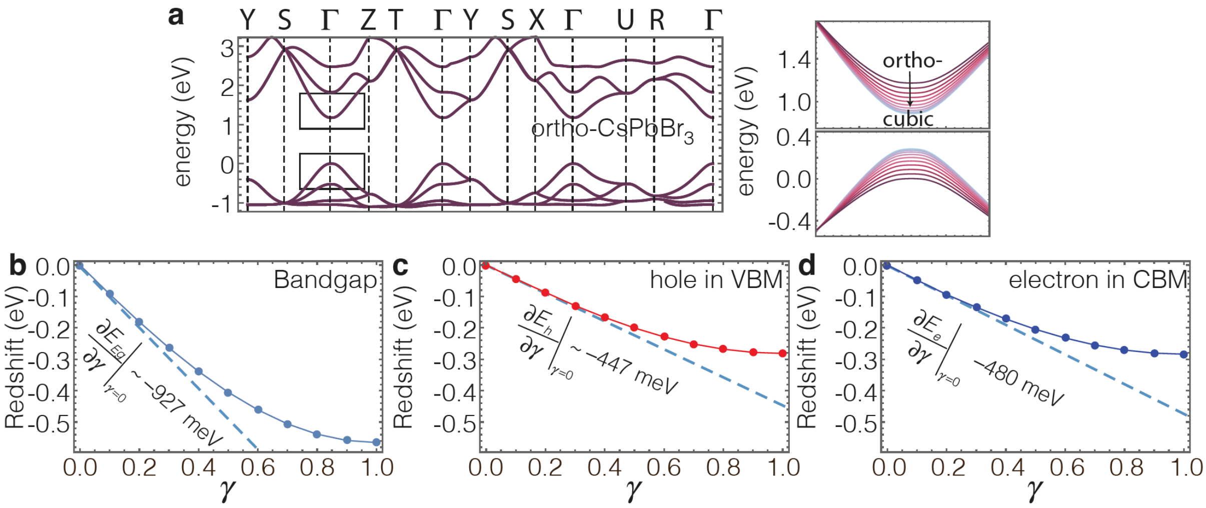

Figure S11. *a) Calculated bandstructure of orthorhombic CsPbBr₃. The bandstructure in regions defined by the black boxes are shown on the right for γ varying from 0 to 1. b-d) Plot of the energy redshift for excitations in CsPbBr₃ as a function of γ, where γ = 0 corresponds to the orthorhombic phase and γ = 1 the cubic phase.*

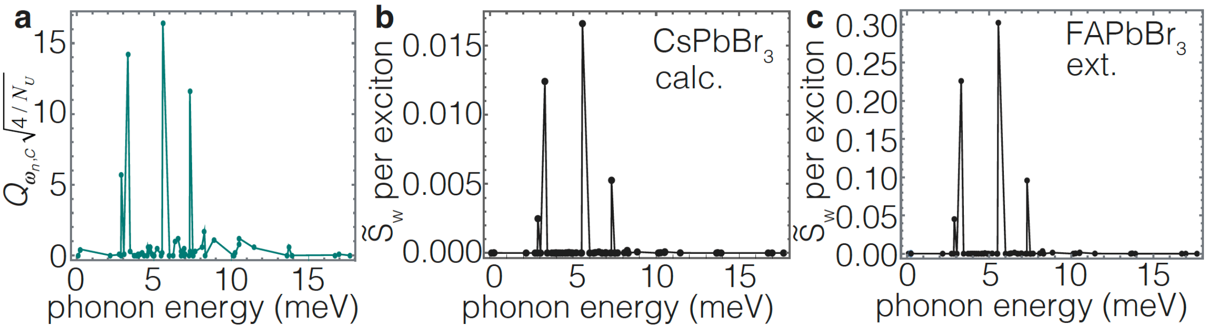

Figure S12. *a) Computed shift of normal coordinates $Q_{\omega_n,c}$ between the orthorhombic and cubic phase of CsPbBr₃. b) The calculated electron-phonon coupling strength $\tilde{S}_{\omega_n}$ to octahedral tilting per exciton in CsPbBr₃ NCs. c) $\tilde{S}_{\omega_n}$ for FAPbBr₃ NCs estimated from the magnitude of the lattice reorganization measured with time resolve electron diffraction.*

Supplementary Note 5: Extracting Electron-Phonon Coupling Strengths from Measured Lattice Reorganization

Coupling to octahedral tilting: From the UED measurements in the main text, we observe a ~3% decrease in the 211 peak intensity in the $FAPbBr_3$ NCs at a fluence of 0.6 mJ/cm^2 corresponding to ~40 excitons. This 3% decrease corresponds to a decrease in the $PbBr_6$ octahedra tilt of ~1.0 degree (**Fig. 2d**) and a $\gamma_m(N_{ex} = 40) \sim 0.07$ (see **Note 4**). As γ_m is linear in N_{ex}, we take $\gamma_m(N_{ex} = 1) \sim 0.0017$. We then can estimate the electron phonon coupling strength resulting from octahedral tilting for a single exciton as $\tilde{S}_{\omega_n} = \left(\gamma_m Q_{\omega_n,c}\right)^2/2$, the results are plotted in **Fig. S12c** (and in **Fig. 3e** of the main text).

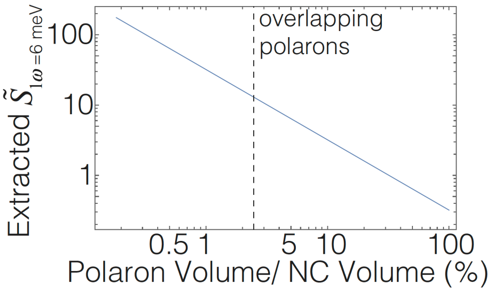

Figure S13. *Plot of the extracted coupling to the 6 meV mode as a result of octahedral tilting as a function of assumed polaron volume. Assuming a polaron volume equal to the NC volume gives provides an excellent match to experimentally measured couplings, while smaller polaron sizes yield unreasonably large coupling strengths.*

We note that the extracted coupling strengths depend upon the assumed polaron radius (through N_U in **eq. S3**), and above we assume all excitons overlap over the entire NC based on the estimated polaron radius in **Note 5**. More generally, without this assumption we can write

$$\gamma_m(N_{ex} = 1) = \gamma_m(N_{ex})\frac{V_{NC}}{N_{ex}V_P} \qquad (S8)$$

where V_{NC} is the volume of the NC and V_P is the polaron volume, which accounts for the fraction of the NC volume contributing to the measured signal. As $Q_{\omega_n,c} \propto \sqrt{V_P}$ is also proportional to the polaron volume, the coupling strengths then scales with the assumed polaron radius $\tilde{S}_{\omega_n} \propto V_P^{-1}$. In **Fig. S13** we plot the extracted coupling to the 6 meV mode as a function of polaron radius. The assumption of $V_P = V_{NC}$ gives an excellent match to previously measured coupling strengths of this mode in similarly sized NCs, where a coupling to a 5 meV mode of ~ 0.15 – 0.35 was estimated for similarly sized NCs.[12] The extracted coupling strength become unreasonably large as the polaron size is reduced below that of the NC volume, even for large overlapping polarons (e.g. $V_P = 1/3\ V_{NC}$ gives S = 1). This is supportive of the assumption that $V_P = V_{NC}$.

Additional Coupling

As discussed in **Supplementary Note 3**, in addition to a photoinduced reduction in tilt, there is likely renormalization of the Pb-Br bonds. Here we assume this renormalization stems from a photoinduced centering of nominally off-centered Pb ions wthin the Br octahedra.

In **Fig. S14a** we plot the fluence dependence of change in the [311] peak extracted from the MeV-UED at a q~3.6 $Å^{-1}$, which is linear in fluence within the range measured. We extract a change of ~2% at a fluence of 0.6 mJ/cm^2.

In **Fig. S14b** we plot the relative increase in the 311 peak intensity as a function of Pb-shifts in the split-cubic structure, averaged over Pb-shifts in the [111] and [100] direction. The change in the 311 with Pb-shifts is non-linear for small equilibrium shifts, which complicates the extraction of $\tilde{S}_{\omega_n}$, as it will depend on the initial equilibrium shift. Previous reports have estimated an equilibrium shift of ~0.13 Å in $FAPbBr_3$.[11] This estimate is in the highly non-linear portion of the scattering intensity scaling curve however (**Fig. S14b**), which is at odds with the linear scaling we observed in **Fig. S14a**. We therefore assume an initial Pb-shift of ~0.2 Å which is then within the region in which the scaling becomes reasonably linear. For extraction of the coupling we then take that 0.6 mJ/cm^2 photoexcitation causes on average a 0.06 Å reduction of the Pb atoms nominally shifted by 0.2 Å. Then, to estimate the coupling, we generate structures with Pb atoms within the *Pnma* structure shifted along the different [111] and [100] directions from the center of the octahedra, and calculate $Q_{\omega_n}(\gamma)$ where γ = 0

corresponds to the equilibrium structure and γ = 1 corresponds to the photoexcited structure (with 0.06 Å reductions in the Pb-shifts). We then average the results over all possible [111] and [100] directions. The coupling is then computed as $\tilde{S}_{\omega_n} = \frac{1}{2}\left(Q_{\omega_n}(1)\right)^2$, giving the coupling strength shown in **Fig. S15**.

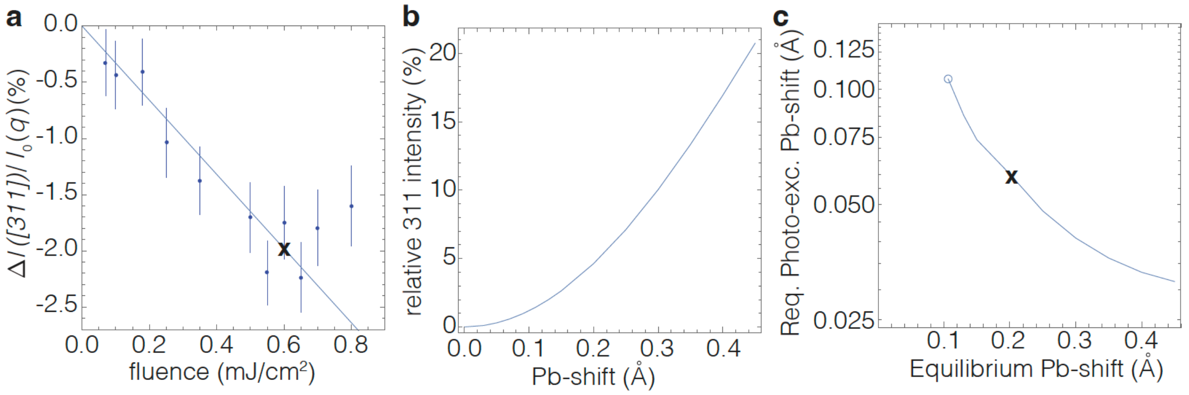

Figure S14. *a) Fluence dependence of the strength of the photo-induced reduction in the 311 peak, extracted from the MeV-UED at a q~3.6 Å^{-1}. Error bars represent 1σ uncertainty. b) relative increase in the 311 peak intensity as a function of Pb-shifts in the split-cubic structure, averaged over Pb-shifts in the [111] and [100] directions. c) Plot of the photo-excited reduction in Pb-shift required to reproduce the ~2% change observed experimentally with 0.6 mJ/cm^2 (x in panel a) as a function of equilibrium Pb-shift in the SC structure. The 'x' marks the equilibrium shift used to estimate EP coupling strengths.*

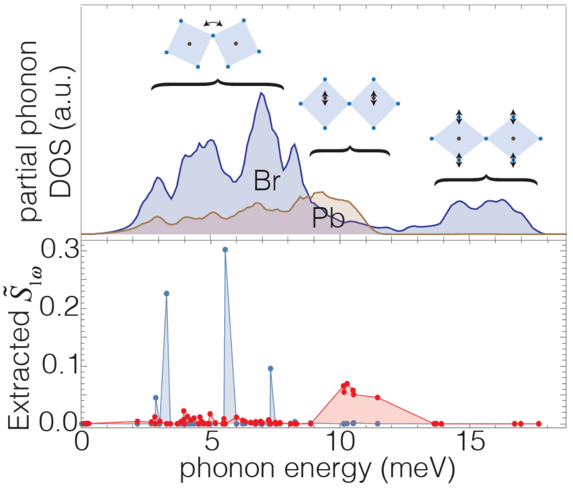

Figure S15. *Extracted coupling resulting from octahedral tilting (blue) and lead off-centering (red)*

Supplementary Note 6: Enhanced Coupling to Low Energy Optical Phonons in Polymorphous $FAPbBr_3$

There are several possible mechanisms which can enhance EP-coupling in the disordered crystal phase. First, the EP-coupling strength $\tilde{S}_{\omega} \propto \omega^{-3}$ (**Supplementary Note 4**), and therefore the softening of a coupled phonon can dramatically enhance the coupling strength. The phonon modes driving Pb-X-Pb bond-angle distortions may be intrinsically soft in the disordered phase.

We show in the following that an entropic contribution to the free energy of glassy structure may also enhance coupling at elevated temperatures. At finite temperatures, the energy of the lattice (**Eq. S4**) should be replaced with the free-energy,

$$G_{tot}(\gamma,T) = E_0 + \sum_n \frac{1}{2}\hbar\omega_n\left(\gamma Q_{\omega_n,c}\right)^2 - TS(\gamma) + N_{ex}\gamma\left.\frac{\partial E_{ex}}{\partial\gamma}\right|_{\gamma=0} + \cdots, \qquad (S9)$$

where T is the temperature and $S(\gamma)$ is the entropy. For small γ,

$$G_{tot}(\gamma,T) = G_0 + \sum_n \frac{1}{2}\hbar\omega_n\left(\gamma Q_{\omega_n,c}\right)^2 - T\gamma\left.\frac{\partial S(\gamma)}{\partial\gamma}\right|_{\gamma=0} + N_{ex}\gamma\left.\frac{\partial E_{ex}}{\partial\gamma}\right|_{\gamma=0} + \cdots, \qquad (S10)$$

which gives a correction in the denominator of the coupling strength (**eq. S6**)

$$\tilde{S}_{\omega_n} = \frac{1}{2}\left(\frac{N_{ex}\left.\frac{\partial E_{ex}}{\partial\gamma}\right|_{\gamma=0}}{\sum_n \hbar\omega_n Q_{\omega_n,c}^2 - T\left.\frac{\partial S(\gamma)}{\partial\gamma}\right|_{\gamma=0}}\right)^2 Q_{\omega_n,c}^2. \quad (S11)$$

We refrain from attempting to determine an expression for $S(\gamma)$, and note that the stabilization of the average cubic phase at high temperatures in LHPs indicate that $\partial S(\gamma)/\partial\gamma$ is finite and positive. From **eq. S11** we can therefore conclude that the coupling strength will increase with increasing temperature as observed in our temperature dependent measurements (**Fig. 4b**) and will depend on the magnitude of $S(\gamma)$. A similar correction to the single mode model in the main text can be similarly done with free energy

$$G_{N_{ex}}(Q) = G_0 + \frac{1}{2}\hbar\omega Q^2 + N_{ex}\left(E_{g0} + \frac{\partial E_g}{\partial Q}Q\right) + T\frac{\partial S}{\partial Q}Q, \quad \text{(S12)}$$

giving

$$Q_{N_{ex}} = \frac{N_{ex}\left(-\frac{\partial E_g}{\partial Q}\right)}{\hbar\omega - T\frac{\partial S}{\partial Q}}, \qquad \text{(S13)}$$

from which the same conclusions can be drawn.

Finally, enhancement of the coupling to the low energy optical modes may stem from their correlation to anharmonic coupling of the rotational modes of the FA ions, such that photoexcitation additionally causes a reorientation of the FA.[17,18]

Supplementary Note 7: FLUPS Measurements on $FAPbBr_3$ and $CsPbBr_3$ NCs

In **Fig. S16a** we plot the total integrated (over energy and time) PL intensity for $CsPbBr_3$ and $FAPbBr_3$ NCs as a function of pump power. While initially increasing with pump power, the total integrated PL saturates at low pump powers, indicative of a rapid decrease in the PL quantum yield (PLQY) with increasing exciton density N_{EX}. We assume here a form

$$PLQY(N_{EX}) = (N_{EX} + N_{EX}^2 + N_{EX}^3) / N_{EX}(1 + N_{EX} + N_{EX}^2 + N_{EX}^3) \quad (S14)$$

Eq. S14 is plot in **Fig. S16b**. This form for the PLQY gives a value of ~1 for low excitation densities ($N_{EX} < 1$) , consistent with the very high PLQY expected in this range, and ~1/ N_{EX} for $N_{EX} >> 1$, i.e. for large N_{EX} we expect on average a single photon out, independent of N_{EX}. This assumed form for the PLQY, we obtain a reasonable agreement to the total integrated PL as a function of pump power (dashed line in **Fig. S16a**). We then use **eq. S14** to compute the relative contribution to the FLUPS signal as a function of N_{EX} for a gaussian pump beam profile. In **Fig. S16c** we plot the relative contribution for 3 pump powers. The conclusion from

these plots is that the dominant contribution to the FLUPS signal will be from weakly (small N_{EX}) excited NCs at the periphery of the beam where the PLQY is high.

In **Fig. S17** we plot the total PL as a function of time for varying pump powers (from 3 to 75 nJ) in $FAPbBr_3$ and $CsPbBr_3$ NCs. The average relaxation time constant from our UED measurements, τ_r, is ~35 ps. For both NCs, multiexciton-decay rates are reasonably fit with a fluence independent time constant of this same value. For $FAPbBr_3$ NCs, a fast decay component with time constant ~10 ps is also observed for high pump intensities. We note that the maximum fluence used in the FLUPS measurements are ~5x more intense than in the UED measurements (~4.5 mJ/cm^2 peak fluence for the 75 nJ power), and speculate that this fast component may stem from very high excitation densities.

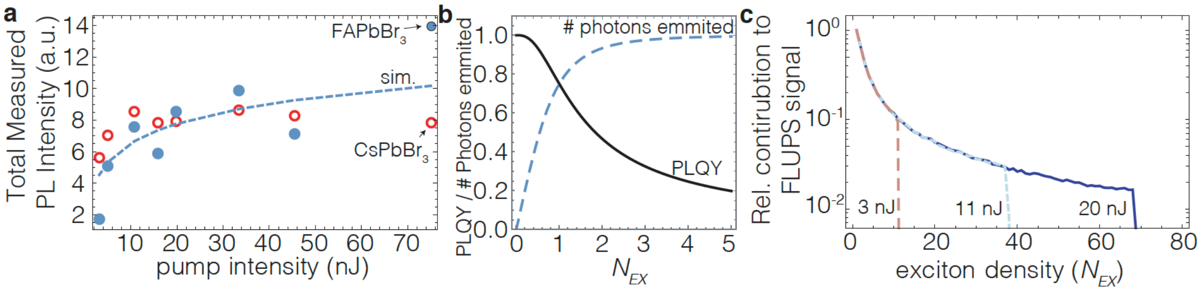

Figure S16. a) Total measured PL intensity, integrated over time and energy, for $FAPbBr_3$ NCs (blue circles) and $CsPbBr_3$ NCs (open red circles). The dashed line shows the simulated intensity assuming a PLQY(N_{EX}) in **eq. S14** and shown in (b). c) Plot of the relative contribution to total FLUPS signal as a function of N_{EX} for various pump intensities. For all pump powers, a majority of the FLUPS signal stems from the PL from weakly excited NCs in the periphery of the beam (where PLQY is high).

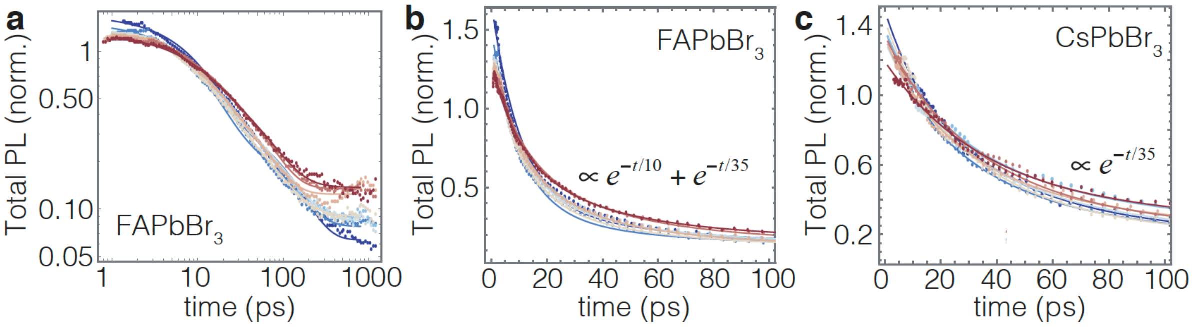

Figure S17. Plot of the total PL as a function of time for varying pump powers (red to blue = 3 to 75 nJ) in $FAPbBr_3$ (a and b) and $CsPbBr_3$ NCs (c). For both NCs, multiexciton-decay rates are reasonably fit with a fluence independent time constant of ~35 ps. For $FAPbBr_3$ NCs, a fast decay component with time constant ~10 ps is also observed for high pump intensities.

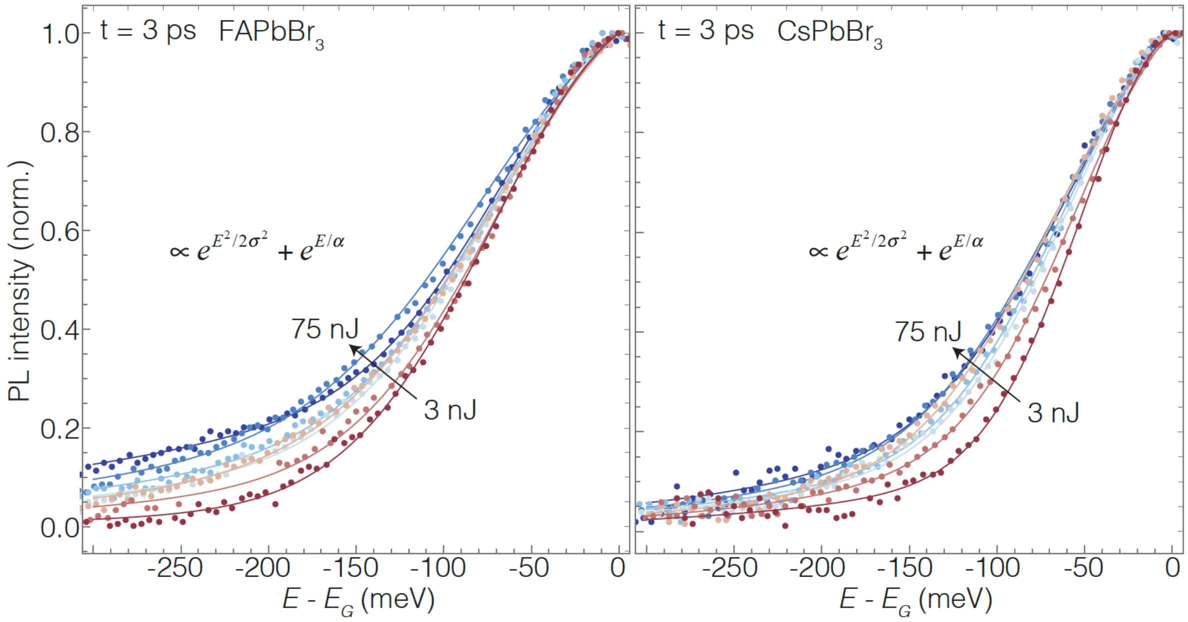

Figure S18. Plots of the normalized emission of $FAPbBr_3$ and $CsPbBr_3$ NCs at 3 ps for varying pump powers (see **Figure 5** in the main text).

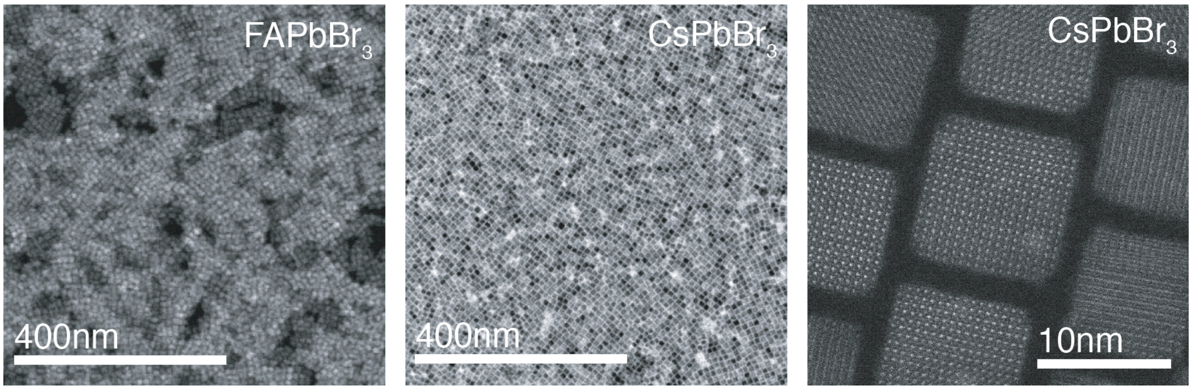

Figure S19. Additional TEM images of nanocrystal samples.

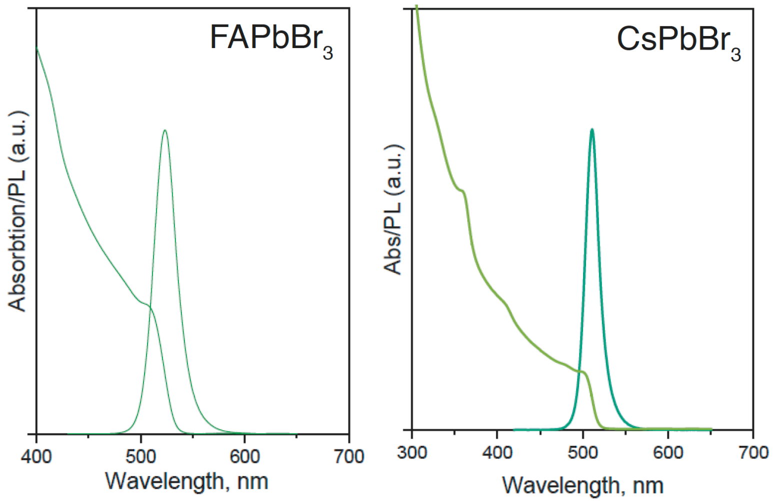

Figure S20. Emission and absorption spectra of nanocrystal samples.

Table S2. Optimized ideal *Pnma* structure of $CsPbBr_3$ in fractional coordinates:

{A, B, C} = {8.50921, 11.8817, 8.30269}

Cs	0.55526	0.25000	-0.01739
Cs	0.44474	0.75000	0.01739
Cs	-0.05526	0.75000	0.48261
Cs	0.05526	0.25000	0.51739
Pb	0.00000	0.00000	0.00000
Pb	0.00000	0.50000	0.00000
Pb	0.50000	0.00000	0.50000
Pb	0.50000	0.50000	0.50000
Br	-0.01199	0.25000	0.07935
Br	0.01199	0.75000	-0.07935
Br	0.51199	0.75000	0.57935
Br	0.48801	0.25000	0.42065
Br	0.21223	0.53967	0.28967
Br	0.78777	0.46033	0.71033
Br	0.28777	0.46033	0.78967
Br	0.71223	0.53967	0.21033
Br	0.21223	-0.03967	0.28967
Br	0.78777	0.03967	0.71033
Br	0.71223	-0.03967	0.21033
Br	0.28777	0.03967	0.78967

References

1. Kirschner, M. S. *et al.* Photoinduced, reversible phase transitions in all-inorganic perovskite nanocrystals. *Nat. Commun.* **10**, 1–8 (2019).
2. Guzelturk, B. *et al.* Dynamic lattice distortions driven by surface trapping in semiconductor nanocrystals. *Nat. Commun.* **12**, 1860 (2021).
3. Diroll, B. T. & Schaller, R. D. Intraband Cooling in All-Inorganic and Hybrid Organic–Inorganic Perovskite Nanocrystals. *Adv. Funct. Mater.* **29**, 1901725 (2019).
4. Elbaz, G. A. *et al.* Phonon Speed, Not Scattering, Differentiates Thermal Transport in Lead Halide Perovskites. *Nano Lett.* **17**, 5734–5739 (2017).
5. Debye, P. Zerstreuung von Röntgenstrahlen. *Ann. Phys.* **351**, 809–823 (1915).
6. Bertolotti, F., Moscheni, D., Guagliardi, A. & Masciocchi, N. When Crystals Go Nano – The Role of Advanced X-ray Total Scattering Methods in Nanotechnology. *Eur. J. Inorg. Chem.* **2018**, 3789–3803 (2018).
7. Cervellino, A., Frison, R., Bertolotti, F. & Guagliardi, A. DEBUSSY 2.0 : the new release of a Debye user system for nanocrystalline and/or disordered materials. *J. Appl. Crystallogr.* **48**, 2026–2032 (2015).
8. Protesescu, L. *et al.* Monodisperse Formamidinium Lead Bromide Nanocrystals with Bright and Stable Green Photoluminescence. *J. Am. Chem. Soc.* **138**, 14202–14205 (2016).
9. Hanusch, F. C. *et al.* Efficient Planar Heterojunction Perovskite Solar Cells Based on Formamidinium Lead Bromide. *J. Phys. Chem. Lett.* **5**, 2791–2795 (2014).
10. Piveteau, L. *et al.* Bulk and Nanocrystalline Cesium Lead-Halide Perovskites as Seen by Halide Magnetic Resonance. *ACS Cent. Sci.* **6**, 1138–1149 (2020).
11. Laurita, G., Fabini, D. H., Stoumpos, C. C., Kanatzidis, M. G. & Seshadri, R. Chemical tuning of dynamic cation off-centering in the cubic phases of hybrid tin and lead halide perovskites. *Chem. Sci.* **8**, 5628–5635 (2017).
12. Cho, K. *et al.* Luminescence Fine Structures in Single Lead Halide Perovskite Nanocrystals: Size Dependence of the Exciton–Phonon Coupling. *Nano Lett.* **21**, 7206–7212 (2021).
13. O'Keeffe, M. & Hyde, B. G. Some structures topologically related to cubic perovskite (E2 1), ReO 3 (D 09) and Cu3 Au (L 12). *Acta Crystallogr. Sect. B Struct. Crystallogr. Cryst. Chem.* **33**, 3802–3813 (1977).
14. Monserrat, B. Electron–phonon coupling from finite differences. *J. Phys. Condens. Matter* **30**, 083001 (2018).
15. Devreese, J. T. Fröhlich Polarons - Lecture course including detailed theoretical derivations -- 10th edition. *ArXiv ID 1611.06122* (2016).
16. Yang, Z. *et al.* Impact of the Halide Cage on the Electronic Properties of Fully Inorganic Cesium Lead Halide Perovskites. *ACS Energy Lett.* **2**, 1621–1627 (2017).
17. Duan, H.-G. *et al.* Photoinduced Vibrations Drive Ultrafast Structural Distortion in Lead Halide Perovskite. *J. Am. Chem. Soc.* **142**, 16569–16578 (2020).
18. Wu, X. *et al.* Light-induced picosecond rotational disordering of the inorganic sublattice in hybrid perovskites. *Sci. Adv.* **3**, e1602388 (2017).
